# Comprehensive Analysis Reveals the Evolution and Pathogenicity of *Aeromonas*, Viewed from Both Single Isolated Species and Microbial Communities

**DOI:** 10.1128/mSystems.00252-19

**Published:** 2019-10-22

**Authors:** Chaofang Zhong, Maozhen Han, Pengshuo Yang, Chaoyun Chen, Hui Yu, Lusheng Wang, Kang Ning

**Affiliations:** a Key Laboratory of Molecular Biophysics of the Ministry of Education, Hubei Key Laboratory of Bioinformatics and Molecular Imaging, Department of Bioinformatics and Systems Biology, College of Life Science and Technology, Huazhong University of Science and Technology, Wuhan, Hubei, China; b Department of Computer Science, City University of Hong Kong, Kowloon, Hong Kong, China; c Clinical Laboratory, Wuhan Children’s Hospital, Tongji Medical College, Huazhong University of Science and Technology, Wuhan, Hubei, China; University of Delhi

**Keywords:** *Aeromonas*, evolution, pathogenicity, pan-genome

## Abstract

The genus *Aeromonas* is a common gastrointestinal pathogen associated with human and animal infections. Due to the high level of cross-species similarity, their evolutionary dynamics and genetic diversity are still fragmented. Hereby, we investigated the pan-genomes of 29 *Aeromonas* species, as well as *Aeromonas* species in microbial communities, to clarify their evolutionary dynamics and genetic diversity, with special focus on virulence factors and horizontal gene transfer events. Our study revealed an open pan-genome of *Aeromonas* containing 10,144 gene families. These *Aeromonas* species exhibited different functional constraints, with the single-copy core genes and most accessory genes experiencing purifying selection. The significant congruence between core genome and pan-genome trees revealed that core genes mainly affected evolutionary divergences of *Aeromonas* species. Gene gains and losses revealed a high level of genome plasticity, exhibited by hundreds of gene expansions and contractions, horizontally transferred genes, and mobile genetic elements. The selective constraints shaped virulence gene pools of these *Aeromonas* strains, where genes encoding hemolysin were ubiquitous. Of these strains, Aeromonas aquatica MX16A seemed to be more resistant, as it harbored most resistance genes. Finally, the virulence factors of *Aeromonas* in microbial communities were quite dynamic in response to environment changes. For example, the virulence diversity of *Aeromonas* in microbial communities could reach levels that match some of the most virulent *Aeromonas* species (such as A. hydrophila) in penetrated-air and modified-air packaging. Our work shed some light onto genetic diversity, evolutionary history, and functional features of *Aeromonas*, which could facilitate the detection and prevention of infections.

**IMPORTANCE** *Aeromonas* has long been known as a gastrointestinal pathogen, yet it has many species whose evolutionary dynamics and genetic diversity had been unclear until now. We have conducted pan-genome analysis for 29 *Aeromonas* species and revealed a high level of genome plasticity exhibited by hundreds of gene expansions and contractions, horizontally transferred genes, and mobile genetic elements. These species also contained many virulence factors both identified from single isolated species and microbial community. This pan-genome study could elevate the level for detection and prevention of *Aeromonas* infections.

## Abstract (precis)

*Aeromonas* has long been known as a gastrointestinal pathogen, yet it has many species whose evolutionary dynamics and genetic diversity had been unclear until now. We have conducted pan-genome analysis for 29 *Aeromonas* species and revealed a high level of genome plasticity exhibited by hundreds of gene expansions and contractions, horizontally transferred genes, and mobile genetic elements. These species also contained many virulence factors both identified from single isolated species and microbial community. This pan-genome study could elevate the level for detection and prevention of *Aeromonas* infections.

## INTRODUCTION

The genus *Aeromonas* comprises a group of Gram-negative bacteria commonly found in aquatic habitats, which have been recognized as opportunistic pathogens associated with a variety of infections in both humans and animals (1–4). More than 35 species of this genus had been sequenced by January 2017, and of these, 29 with complete genomes were available in public databases. As these strains are isolated and studied, the presence of virulence genes associated with human and animal infections, such as *aerA* and *hlyA*, have been examined (5, 6). Four *Aeromonas* species, A. hydrophila, A. veronii, A. caviae, and A. dhakensis have been reported to be responsible for a wide range of clinical infections (3). In particular, *A. hydrophila*, *A. caviae*, and A. veronii, which share many biochemical characteristics, are important pathogens that can cause necrotizing fasciitis (7–9). In addition, as the first to be isolated from stools of children with diarrhea, *A. dhakensis* is more virulent than *A. veronii*, *A. caviae*, and *A. hydrophila* (10, 11). Hence, it is necessary to make systematic comparisons on the species level and gene level to decipher the characteristics of this genus.

The *Aeromonas* species are recognized for their high-level homogeneity, which makes it difficult to identify these species only from phenotypes (11, 12). For example, despite the fact that *A. dhakensis* has the typical *Aeromonas* characteristics, such as motile Gram-negative bacilli, capable of reducing nitrate to nitrite, and able to undergo both respiration and fermentation, it is often misidentified as *A. hydrophila* (11, 12). In addition, marker genes, such as 16S rRNA, *rpoD* and *gyrB* genes, are unreliable for distinguishing closely related *Aeromonas* species (13) or in the identification of *Aeromonas* to the species level due to their low heterogeneity (14). For example, since 16S rRNA does not unambiguously distinguish *A. dhakensis* from *A. caviae* (15), this inaccurate species identification may lead to incorrect assessment of the actual pathogenic potential (11). Accurate taxonomy can improve our knowledge about the epidemiological distribution and pathogenic potential of human pathogens. Although many species have been sequenced, phylogenetic analyses of total conserved genes within the *Aeromonas* genus remain limited. Analysis of core genes can provide deeper insights into the evolution and phylogeny of *Aeromonas* because of their high resolution in distinguishing closely related species (16). Therefore, it is necessary to have a deeper understanding of the evolution of *Aeromonas* based on extensive analysis of whole-genome sequences from both single isolated species and microbial communities.

The pathogenicity of *Aeromonas* can be attributed to a broad range of virulence factors (17, 18). It has already been reported that *Aeromonas* species have caused many infections around the world (19–21). In addition, as previously reported, many extracellular proteins, including hemolysins, endotoxins, and adherence factors, are associated with the pathogenesis of *Aeromonas* (5, 22, 23). Several secretion mechanisms that contribute to the export of virulence genes in *Aeromona*s, such as type II, III, and VI secretion systems (T2SS, T3SS, and T6SS) have also been identified (24, 25). In addition, efforts have been made to distinguish the pathogenic potential of *A. hydrophila*, *A. veronii*, and *A. caviae* (26). Some mobile genetic elements (MGEs) that carry virulence or antibiotic resistance genes, such as plasmids and insertion sequences, have been reported to be involved in the genomic plasticity of *Aeromonas* (27, 28). However, though many virulence factors of *Aeromonas* have been identified (22), little is known about their evolutionary dynamics, especially dynamic changes in the microbial community. It would be very natural and quite intriguing to ascertain the evolution of *Aeromonas* strains living in microbial communities. Pan-genome analysis is an effective method to evaluate genomic diversity (29, 30). However, though numerous whole-genome sequences of *Aeromonas* species are already available in public databases, there are few studies on the pan-genomes of these species, resulting in a lack of understanding of virulence factors in *Aeromonas* species. At this time, some studies have looked into the virulence of *Aeromonas* (6, 31), but the recent pan-genome study was carried out in only three species (*A. hydrophila*, *A. veronii*, and *A. caviae*). Thus, the recently available whole-genome sequences of *Aeromonas* species allow for a large-scale pan-genome analysis.

Hence, to gain deeper insight into the genome of *Aeromonas* at the species level, we conducted a pan-genome analysis for 29 different *Aeromonas* species isolated from diverse ecological niches, focusing on the genetic diversity, phylogenetic relationships, and evolutionary trends of virulence factors. We deduced phylogenetic relationships and evolutionary trends of *Aeromonas*, detected the recently evolved genes via gene gain and loss, characterized the virulence and antibiotic resistance, and emphasized the influence of gene gain and loss in the evolution of virulence in *Aeromonas*. In addition, we have extended the scope of pathogenicity analysis for *Aeromonas*, as we attempted to identify the dynamics of its virulence factors in the microbial community setting. The present study highlighted the virtue of pan-genome analysis and microbiome analysis in inferring evolutionary cues for *Aeromonas* species, which revealed genomic diversity in the pathogenic potential of *Aeromonas*.

## RESULTS AND DISCUSSION

### Pan-genome construction and analysis.

**FIG 1:**
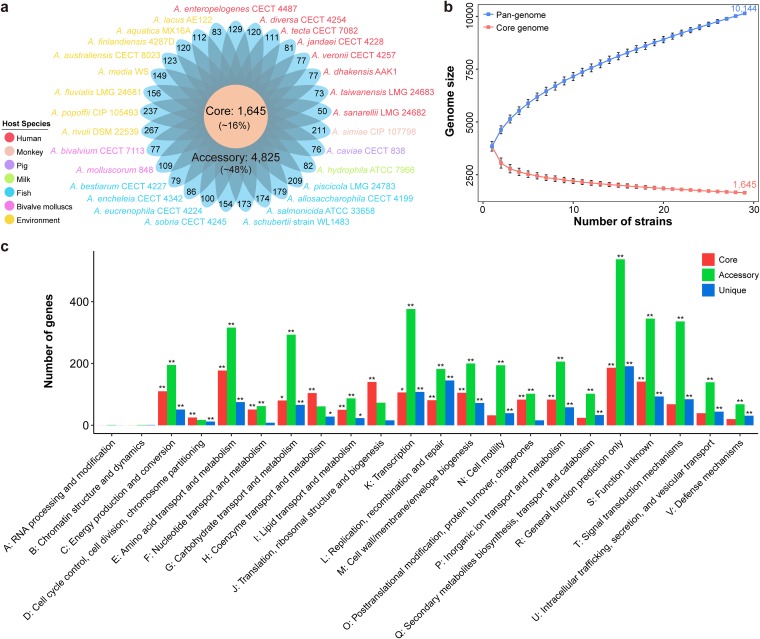
Pan-genome structure and function of *Aeromonas*. (a) Distributions of core genes, accessory genes, and unique genes. Strains are colored according to their isolation sites. (b) Gene accumulation curves for the pan-genome (blue) and core genome (red). The cumulative sizes of the pan-genome and core genome were calculated by selecting strains without replacement in random order 1,000 times and then calculating the mean size of each sampling point. Error bars indicate one standard deviation from the mean. (c) Distribution of COG categories in *Aeromonas* core, accessory, and unique genome. The numbers of genes assigned by COG categories in the core genome (red bars), accessory genome (green bars), and unique genome (blue bars) are shown. Genes without homologs are not included in the statistics. *, Fisher’s exact test *P* value < 0.05; **, Fisher’s exact test *P* value < 0.01.

To characterize the genomic composition among different *Aeromonas* species, 29 genomes isolated from bacteria found in different ecological niches with the highest level of completeness for a species were used for pan-genome analysis (see Table S1 in the supplemental material). In total, these strains contained 10,144 orthologous groups (defined as gene families), which were organized into core, accessory, and unique genes (Table S2). We found that 1,645 gene families (16%) shared by all strains constituted the core genome, while the remaining 8,499 (84%) were variably represented genes. This variable genome of 29 strains in the *Aeromonas* genus made up a substantial portion (84%) of the pan-genome of the genus as a whole, suggesting a high degree of genetic variation. Among these variable gene families, 3,674 gene families specific to a single strain constituted unique genomes, and the remaining 4,825 gene families present in more than one strain but not in all strains belonged to the *Aeromonas* accessory genome (Table S2 and Fig. 1a). Remarkably, the core genome comprised 1,601 orthologous (single-copy) gene families and 44 paralogous (multicopy) gene families. In addition, the distribution of unique genes in *Aeromonas* was diverse, varying from 50 to 267 (Fig. 1a), in which A. rivuli DSM 22539 clearly stood out by possessing the highest number (267 genes) of unique genes, whereas A. sanarellii LMG 24682 harbored the minimum number (50 genes) of unique genes. Although the genome of A. fluvialis LMG 24681 was the smallest (32), it contained 156 unique genes. The considerable number of accessory and unique genes further emphasized the heterogeneity of the genus, implying very high genome plasticity.

10.1128/mSystems.00252-19.5 TABLE S1 Bacterial strains and sources used for the genomic comparison of 29 *Aeromonas* species in this study. The genomic features and geographical origins of 29 *Aeromonas* genomes used in the study were shown. Download Table S1, PDF file, 0.02 MB. Copyright © 2019 Zhong et al. 2019 Zhong et al. This content is distributed under the terms of the Creative Commons Attribution 4.0 International license.

10.1128/mSystems.00252-19.6 TABLE S2 Summary of homologous gene identified for 29 *Aeromonas* strains by OrthoMCL. The total numbers of clusters of homologous genes and the numbers of core, accessory, and strain-specific unique genes for 29 *Aeromonas* are shown. Download Table S2, PDF file, 0.03 MB. Copyright © 2019 Zhong et al. 2019 Zhong et al. This content is distributed under the terms of the Creative Commons Attribution 4.0 International license.

On the basis of the gene accumulation curve, when encompassing 29 strains, *Aeromonas* exhibited an open pan-genome structure whose size was estimated to be 10,144 nonredundant genes, which tended to rise progressively (Fig. 1b). In order to estimate gene diversity with the number of *Aeromonas* species, we calculated the diversity by estimating the number of genes based on the Chao1 estimator, an abundance-based nonparametric estimator, in which a higher value means a greater diversity (33). The rarefaction curve of Chao1 was not saturated, and Chao1 values varied within 17,574 ± 361 for the 29 strains (see Fig. S1a in the supplemental material). This pan-genome was larger than previously reported (26), indicating that the increase in genomic data size would result in the expansion of pan-genome sizes and greater genomic diversity. Rarefaction analysis of the number of nonredundant genes against the number of strains showed that the genes shared by more than one strain (nonunique genes) were almost constant with approximately 29 strains, while unique genes were still increasing in the presence of 29 strains (Fig. S1b). The number of nonunique genes in the *Aeromonas* strains covered 63.78% of the total nonredundant genes. These results provide further evidence for the high variability of the unique parts of the genome and suggest that sequencing of additional strains would result in a higher number of unique genes. In contrast to the pan-genome, the estimated core genome size of the 29 strains included in our analysis has gradually decreased and has not approached a plateau (Fig. 1b). We measured the number of core genes as a function of the number of strains using the power regression model. Our results showed that in the regression model, the core genes as a function of the number of strains was well described by a decaying power function, with a fitted exponent of −0.236 ± 0.004 (Fig. S1c). These results indicated that both the core genome and the pan-genome were influenced by the inclusion of newly sequenced strains.

10.1128/mSystems.00252-19.1 FIG S1 Gene accumulation curves for the 29 *Aeromonas* strains. (a) Richness predicted by Chao1. (b) The number of detected genes is plotted against the number of strains. The number of the genes shared by all and by >1, >2, >3, >4, and >5 of 29 strains are shown. (c) Number of core genes as a function of the number of strains. The violet line is a fit with a decaying power function *y* = *A*/*x^B^*, with A = 3,729.985 ± 36.838 and B = 0.236 ± 0.004. (d) Putative functions based on Gene Ontology (GO) annotation of core, accessory, and unique genome. The core genome (red bars), accessory genome (green bars), and unique genome (specific genes) (blue bars) count for the most abundant 40 GO categories are graphed. *, Fisher’s exact test *P* value < 0.05; **, Fisher’s exact test *P* value < 0.01. Download FIG S1, TIF file, 2.0 MB. Copyright © 2019 Zhong et al. 2019 Zhong et al. This content is distributed under the terms of the Creative Commons Attribution 4.0 International license.

### Functional characterization of the *Aeromonas* pan-genome.

An open pan-genome helps the species respond to diverse environments. To gain insight into the functional features of the pan-genome, we characterized functions of the core, accessory, and unique genes by searching the Clusters of Orthologous Groups (COG). A high proportion (59%) of the pan-genome was poorly characterized (categories “general function prediction only,” “function unknown,” and “no homologs identified”), since the proteins encoded by these genes were either functionally unknown or did not have homologs outside the genus. The core genome conferred an extensive functional repertoire that have fundamental roles in the maintenance of primary cellular process, including metabolism (such as metabolism of amino acids [Fisher’s exact test *P* value < 0.001], coenzymes [Fisher’s exact test *P* value < 0.001], nucleotides [Fisher’s exact test *P* value < 0.001], inorganic ions [Fisher’s exact test *P* value < 0.001], and lipids [Fisher’s exact test *P* value < 0.001]) and information storage and processing (such as translation [Fisher’s exact test *P* value < 0.001] and replication [Fisher’s exact test *P* value = 0.003]) (Fig. S2a and Fig. 1c). Genes assigned to “transcription” (376 genes, Fisher’s exact test *P* value < 0.001), “amino acid transport and metabolism” (316 genes, Fisher’s exact test *P* value < 0.001), “carbohydrate transport and metabolism” (293 genes, Fisher’s exact test *P* value < 0.001), and “signal transduction mechanisms” (336 genes, Fisher’s exact test *P* value < 0.001) were prominently represented in the accessory component of the pan-genome (Fig. S2a and Fig. 1c). The unique genome had a high proportion (2,673/3,674) of genes with no identified homologs. The remaining proportion (1,001/3,674) unique genes carried diverse functions such as “transcription” (108 genes, Fisher’s exact test *P* value < 0.001), “replication, recombination and repair” (145 genes, Fisher’s exact test *P* value < 0.001), and “signal transduction mechanisms” (84 genes, Fisher’s exact test *P* value < 0.001). The number of genes encoding functions “cell cycle control, cell division, chromosome partitioning,” “coenzyme transport and metabolism,” and “translation, ribosomal structure and biogenesis” were higher in the core genome than in the accessory and unique genome (Fig. 1c). In addition, several categories had a higher percentage in the core genome but were less represented in the accessory or unique genome. For example, the proportion of the core genome assigned to “defense mechanisms” (1.21%) was actually higher than those in the unique genome (0.84%). Similarly, the core genome had a higher percentage of “replication, recombination and repair” genes (4.92%) than accessory (3.77%) and unique (3.95%) genomes (Fig. S2a). The category “replication, recombination and repair” contained genes involved in mobile elements (transposase, recombinase, and integrase genes) (34), indicating the presence of potential horizontal gene transfer (HGT) events. In particular, strains such as A. media WS, *A. fluvialis* LMG 24681, A. allosaccharophila CECT 4199, and A. schubertii strain WL1483 possessed more unique genes with “replication, recombination and repair” function (Fig. S2c), indicating potential HGT events.

10.1128/mSystems.00252-19.2 FIG S2 Distribution of COGs for each gene set. (a) The graph shows the percentage of gene composition for each COG in each gene set. Each bar represents a gene set, and each color represents a COG category. (b) Statistics of the number of gene families in each gene set. Each bar represents a gene set identical to the gene set in panel a, and the number of gene families is shown. (c) Comparison of COGs for strain-specific unique genes among each strain. The number of unique genes in COGs for each strain is shown. Download FIG S2, TIF file, 2.6 MB. Copyright © 2019 Zhong et al. 2019 Zhong et al. This content is distributed under the terms of the Creative Commons Attribution 4.0 International license.

We also conducted a Gene Ontology (GO) analysis to characterize gene functions according to biological process, molecular function, and cellular component. The core genes were enriched in “ion binding” (273 genes, Fisher’s exact test *P* value < 0.001), “biosynthetic process” (246 genes, Fisher’s exact test *P* value < 0.001), “cellular nitrogen compound metabolic process” (163 genes, Fisher’s exact test *P* value < 0.001) (Fig. S1d). We detected 16 core genes common to the genus *Aeromonas* that did not have homologous genes in any other sequenced bacteria. They were genus-specific genes and encoded hypothetical proteins (Table S3). The accessory genes were enriched in “DNA binding” (339 genes, Fisher’s exact test *P* value < 0.001), “transport” (259 genes, Fisher’s exact test *P* value < 0.001), and “oxidoreductase activity” (231 genes, Fisher’s exact test *P* value = 0.004) (Fig. S1d). A high proportion (2,947/3,674) of unique genes had no known functions. A total of 347 unique genes were associated with categories such as “DNA binding,” “ion binding,” and “DNA metabolic process” (Fig. S1d). Varied functions conferred by unique genes appeared to be required by members of the genus to respond to the environmental changes, and the high number of uncharacterized unique genes in the *Aeromonas* pan-genome deserve further attention.

10.1128/mSystems.00252-19.7 TABLE S3 Summary of 16 predicted genus-specific genes of *Aeromonas*, 50 predicted horizontally transferred genes in core genome and 46 predicted virulence genes present in all 29 strains. Download Table S3, PDF file, 0.5 MB. Copyright © 2019 Zhong et al. 2019 Zhong et al. This content is distributed under the terms of the Creative Commons Attribution 4.0 International license.

### Phylogenetic analysis of the *Aeromonas* pan-genome.

**FIG 2:**
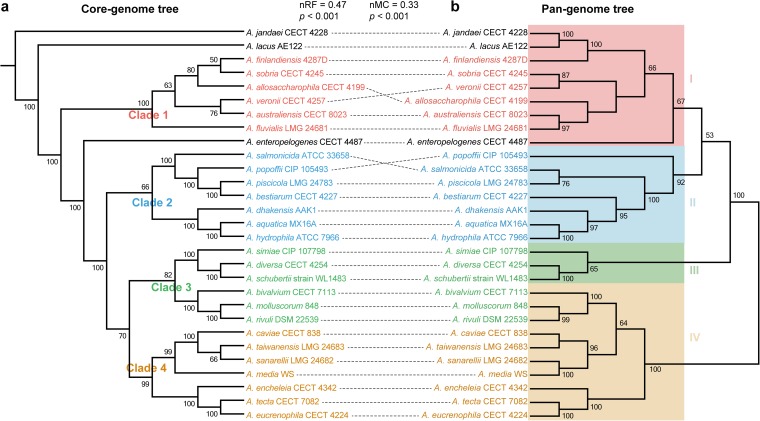
Comparison of two phylogenetic trees constructed using the core genome and pan-genome, respectively. (a) Phylogenetic tree based on the concatenated 1,601 single-copy genes. The tree is divided into four main clusters (clades 1, 2, 3, and 4) based on the topological structure and evolutionary distance. (b) A pan-genome tree based on presence or absence of the gene in the pan-genome. The tree is also divided into four clusters (clades I, II, III and IV) according to topological structure and evolutionary distance. Normalized Robinson-Foulds (nRF) and normalized matching cluster (nMC) scores are used to measure the congruence of the two trees. The bootstrap values are displayed on the trees.

Phylogenetic relationships of strains as seen by constructing phylogenetic trees by using the concatenated core genes allow for a high resolution and establishments of different intrageneric complexes (16). Here, we constructed a core genome phylogenetic tree of 29 *Aeromonas* strains (defined as the core genome tree) based on 1,601 concatenated single-copy core genes. According to the topological structure and evolutionary distance, we divided the tree into four main clusters (clades 1, 2, 3 and 4 [Fig. 2a]), in which three strains A. jandaei CECT 4228, A. lacus AE122, and A. enteropelogenes CECT 4487 diverged independently from other members. *A. jandaei* CECT 4228 was the first or the most ancient divergence of these 29 *Aeromonas* strains. Strains from different isolated sources and with different disease status and geographical origins intermingled in each clade (Fig. S3a), suggesting potential spread and transmission between different ecological niches.

10.1128/mSystems.00252-19.3 FIG S3 Geographical origin of strains and organization of the virulence gene clusters in *Aeromonas*. (a) Phylogenetic relationships with strains by geographical origin. The tree based on single-copy core genes is shown on the left. Source categories (isolated source, healthy/diseased, and geographical origin) are graphed. For the healthy/diseased status, “Undetermined” indicates that the strains were from animals or humans with unknown status, and “Not applicable” indicates that the strains were from environment, milk, or bivalve molluscs. (b) Presence/absence of genes or gene clusters related to toxin, T6SS, iron uptake, type I fimbriae, and Flp type IV pili. Colored arrows represent the presence of a gene or gene cluster within a genome, while a white arrow represents the absence of a gene or gene cluster. Download FIG S3, TIF file, 2.0 MB. Copyright © 2019 Zhong et al. 2019 Zhong et al. This content is distributed under the terms of the Creative Commons Attribution 4.0 International license.

To quantify the correlation between phylogeny and genome composition, we also constructed a dendrogram based on the presence or absence of the 10,144 nonredundant genes (defined as the pan-genome tree). We also obtained the same major clades (clade I, clade II, clade III, and clade IV) according to topological structure and evolutionary distance (Fig. 2b). However, there was discordance in the branching order and phylogenetic placement of these cluster groups among these species between the core genome tree and the pan-genome tree. The four clades exhibited similar species composition with the four clades in the core genome tree, but a discrepancy was noted with the subclade composed of A. bivalvium CECT 7113, A. molluscorum 848, and *A. rivuli* DSM 22539. It displayed a closer relationship to the subclade consisting of A. simiae CIP 107798, A. diversa CECT 4254, and A. schubertii strain WL1483 across core genes (in clade 3), which appeared as sister taxa to each other. However, it shared closely phylogenetic relatedness to the subclade formed by *A. caviae* CECT 838, A. taiwanensis LMG 24683, A. sanarellii LMG 24682, and *A. media* WS (in clade IV) in the pan-genome tree. In addition, in contrast to the core genome tree, phylogenetic positioning of *A. jandaei* CECT 4228, *A. lacus* AE122, and *A. enteropelogenes* CECT 4487 were within clade I in the pan-genome tree (Fig. 2b).

To determine topological correlation between these two phylogenetic trees, we measured the congruence by using normalized Robinson-Foulds (nRF) (35) and normalized matching-cluster (nMC) (36) values ranging from 0 to 1. A score (both nRF and nMC) of 0 indicated that the trees under investigation are congruent, whereas a score of 1 indicates no congruence, and lower nRF and nMC scores indicate a high level of congruence between two trees (37). Comparing phylogeny based on the core genome to that based on the pan-genome, we found that phylogenetic relationships for genes in the core genome bore a high resemblance to the relationships among whole gene content (nRF = 0.47, *t* test *P* value < 0.001; nMC = 0.33, *t* test *P* value < 0.001) (Fig. 2b), despite the occurrence of a large number of variable genes. The relative positions of the *A. allosaccharophila* CECT 4199, *A. veronii* CECT 4257, A. salmonicida ATCC 33658, and A. popoffii CIP 105493 species differed between the two trees. *A. sobria* CECT 4245 and *A. veronii* CECT 4257 segregated under a common node in the pan-genome tree but segregated together under distinct nodes in the core genome tree. *A. salmonicida* ATCC 33658 and A. piscicola LMG 24783 clustered together in the pan-genome tree, which suggests that there might be certain interspecies similarities in the gene repertoire between these species. However, such similarities could not hinder lineage-specific segregation. These results suggested that phylogenetic relationships among *Aeromonas* strains were mainly affected by the content of shared genes, but the variable genes still accounted for an important proportion of phylogenetic signals, and genetic diversity was of great significance in evolution.

### Evolution of *Aeromonas* core and accessory genomes.

**FIG 3:**
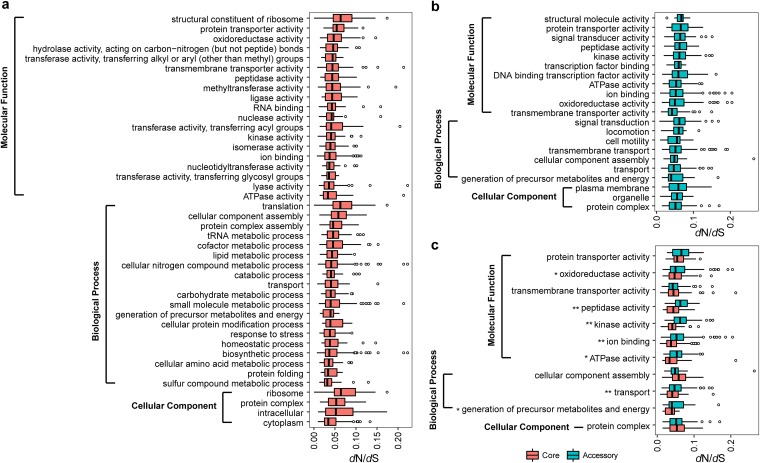
Selection constraints on gene functions in *Aeromonas* core and accessory genome. (a and b) Distribution of selection pressure (*dN*/*dS*) with enriched GO functions in the single-copy core genome (a) and the accessory genome (b). (c) Selection pressure (*dN*/*dS*) with functional categories of the GOs most enriched in the core genome compared with those of the accessory genome are shown. *, *t* test *P* value < 0.05; **, *t* test *P* value < 0.01.

To pinpoint the critical genetic functions changes within the genus *Aeromonas*, we characterized signatures of evolution of 1,601 single-copy core genes and 2,838 accessory genes (genes shared by more than four strains) measured by their ratios (*dN*/*dS*) of nonsynonymous versus synonymous substitution rates. The *dN*/*dS* ratios of less than 1 of all single-copy core genes (average *dN*/*dS* = 0.054 ± 0.03) and 2,834 accessory genes (average *dN*/*dS* = 0.081 ± 0.053) strongly suggested a predominant action of purifying selection within the core genome and most accessory genomes across strains of the different *Aeromonas* species. The accessory genome exhibited great proportion evolving under purifying selection, in addition to four genes (*pilB*, *RS01775*, *RS01685*, and *RS00505*) that we identified as positively selected. Consistent with the fact that housekeeping genes are expected to evolve under strong purifying selection, our analysis revealed that there have been amounts of purifying selection pressure on core and accessory genome components during the diversification of the genus *Aeromonas* and that this selection pressure differs among GO categories. Moreover, the purifying selection pressure on these core genes was stronger than that on accessory genes (*t* test *P* value < 0.001), and there were significant differences in selection pressure among GO functions (Kruskal-Wallis test *P* value < 0.001). The varied functions conferred by single-copy core genome were necessary by members of the genus to handle the housekeeping function. The *dN*/*dS* ratio of each function revealed functional constraints of *Aeromonas* core genes, which evolved by strong purifying selective constraints that would maintain a stable and adapted genomic core. In order to compare the degree of constraint of each function, we merged all the genes associated with their GO categories to compare the selection pressure of each function. Genes involved in the “ATPase activity,” “sulfur compound metabolic process,” and “cytoplasm” functions exhibited significant stronger evolutionary constraints than “structural constituent of ribosome,” “translation,” and “ribosome” in the core genome (*t* test *P* value < 0.001) (Fig. 3a). Moreover, in the accessory genome, the “transmembrane transporter activity,” “generation of precursor metabolites and energy,” and “protein complex” underwent relatively stronger constraints than other functions in molecular function, biological process, and cellular components, respectively (*t* test *P* value < 0.001). Also, the “structural molecule activity,” “signal transduction,” and “plasma membrane” genes evolved under relatively relaxing purifying selection compared to genes with other functions in molecular function, biological process, and cellular component, respectively (*t* test *P* value = 0.007) (Fig. 3b). For the GO functions that core and accessory genes both enriched, we compared the evolutionary rates whose core genes showed significantly stronger evolutionary constraints than accessory genes (*t* test *P* value < 0.001), further demonstrating that the core gene was more stable. Furthermore, we found that the core genes encoding the functions of “oxidoreductase activity,” “peptidase activity,” “kinase activity,” “ion binding,” “ATPase activity,” “transport,” and “generation of precursor metabolites and energy” were under significantly stronger purifying selection pressure than the corresponding accessory genes (*t* test *P* value < 0.05) (Fig. 3c).

### Gene gain and loss for the *Aeromonas* pan-genome.

**FIG 4:**
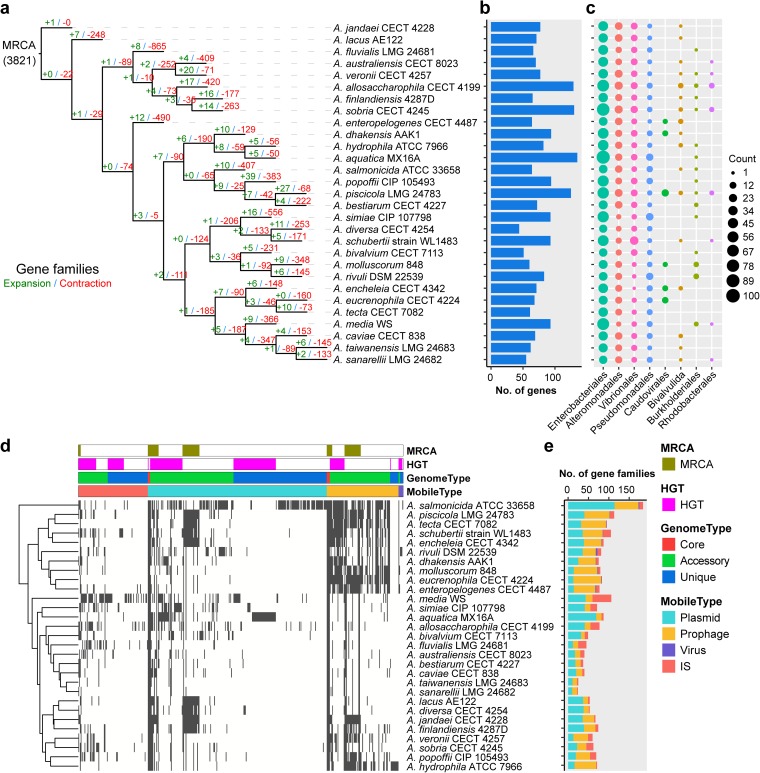
Gene gain and loss reconstruction of *Aeromonas*. (a) Expansion and contraction of gene family in each evolutionary branch. The expansion or contraction of the gene family was estimated by CAFÉ. The number of expanded (green) or contracted (red) genes are shown in each branch. MRCA, most recent common ancestor, which was estimated by CAFÉ. (b) Distribution of horizontally transferred genes acquired in each strain. (c) The eight potential donor bacterial taxa providing donor genes for horizontal transfer. Each circle represents the number of acquisitions. (d) Hierarchical clustering using presence/absence pattern of mobile elements was performed, with the generated dendrogram shown to the left. A gray bar indicates the presence of a gene. (e) Composition and distribution of mobile elements. The rows in panels a, b, and c represent strains and are in the same order. The rows in panels d and e represent strains and are in the same order, and the color legend applies to both panels d and e.

Gene family expansion and contraction are characterized by changes in gene number within gene families (38). The extensive copy number variation and a rapid accumulation of mutations expanded the size of gene families, while the reduction of genes contracted the size of gene families (39). These changes in the size of gene families can play a role in shaping the morphological, physiological, and metabolic differences among species (40). To explore the evolutionary flexibility that may have driven the diversification of *Aeromonas* into present-day species, we used CAFÉ (41) to infer gene family expansion and contraction compatible with the phylogenetic tree of these 29 *Aeromonas* species from the gene repertoire of their most recent common ancestor (MRCA) to the widespread current species. By comparing 29 *Aeromonas* species, 3,821 gene families were estimated to be present in the ancestral genome, of which 58% (2,216 out of 3,821) was identified significantly changed in gene family size (Fig. 4a), suggesting that their genetic repertoires were varied and plastic. The evolutionary flexibility of these *Aeromonas* genomes was evident in the determinations of gene gain and loss on each of the respective lineages. Determinations of the number of genes expanded and contracted on each branch showed considerable variation, and the contraction was considerably greater than expansion, which was particularly evident on external branches. The fact that gene contraction remained greater than expansion suggests that loss of function has an important role in functional evolution and reflects probable vertical descent in most known genomes. Previous studies have reported that gene gain and loss are two contributors to functional change (42). Importantly, the expanded gene families by external branches were enriched in “cell wall/membrane/envelope biogenesis” (Fisher’s exact test *P* value = 0.03) and “cell motility” (Fisher’s exact test *P* value < 0.001), functions related to cellular processes and signaling (Fig. S2a). These results suggest that environmental stress has a potential impact on these strains, making it possible for them to adapt to different environments by requiring cell wall alterations. In addition, these contractions were involved in “signal transduction mechanisms” (Fisher’s exact test *P* value < 0.001), “amino acid transport and metabolism” (Fisher’s exact test *P* value < 0.001), and “transcription” (Fisher’s exact test *P* value < 0.001) (Fig. S2a). The genetic diversity that accompanied these expansions or contractions contributed to functional diversification of *Aeromonas*. In addition, high levels of gene expansion and contraction were evident, even for closely related species. For example, *A. dhakensis* AAK1 expanded 10 genes and contracted 129 genes since it diverged from *A. hydrophila* ATCC 7966. Nevertheless, gene expansion and contraction were extremely rare in the *A. jandaei* CECT 4228 branch, which might be more primitive. Our results revealed large changes in the size of gene families, and they were represented in some functional categories, suggesting that these variations could play a major role in shaping functional differences among species.

Horizontal gene transfer is the movement of genetic material that integrates newly acquired DNA into the genomes of recipients by recombination or insertion (43). HGT is the driver for bacterial genetic innovation and speciation, and strains with newly acquired genes could gain a new physiological function, which is crucial for rapid adaptation to changing environmental conditions (44, 45). Thus, we identified HGTs and quantified their effect on the composition and structure of the pan-genomes of 29 *Aeromonas* strains. We have also source tracked these identified HGTs, especially for those recently acquired HGT genes. We obtained 625 gene families that would be widespread via HGT in these strains (Fig. S2b), of which 249 were unique genes. These putatively transferred genes modulating gene inventory were mainly from *Enterobacteriales*, *Alteromonadales*, and *Vibrionales* (Fig. 4c) and mainly involved in functions such as “replication, recombination and repair” (Fisher’s exact test *P* value < 0.001) and “energy production and conversion” (Fisher’s exact test *P* value < 0.001, Fig. S2a). The *A. aquatica* MX16A isolated from water seems to have experienced a high number of HGT events among the analyzed species. The *A. allosaccharophila* CECT 4199, *A. sobria* CECT 4245, and A. piscicola LMG 24783 isolated from fish also contained a larger number of genes obtained by horizontal transfer, which were 129, 130, and 125, respectively. Our results showed that HGT has contributed to both the core and variable elements of the *Aeromonas* pan-genome. We also found that 50 core genes had potential HGT events (Table S3) during the diversification of the genus *Aeromonas*, which were mainly involved in functions of “oxidoreductase activity” (Fisher’s exact test *P* value < 0.001), “ion binding” (Fisher’s exact test *P* value = 0.004), and “translation” (Fisher’s exact test *P* value < 0.001). This was consistent with a previous report that extensive HGT has been documented in the core genome (46).

The presence of HGT can often be attributed to the uptake of DNA from the environment and the existence of the action of mobile genetic elements (MGEs) (45, 47). These MGEs, including a series of insertion sequences (IS), plasmids, prophages, and viruses, can mediate the movement of DNA and facilitate the transmission of genetic material between different individuals, leading to the rapid acquisition of new functional genes between bacteria (38, 40). Here, to infer the influence of the MGEs, we identified 421 gene families associated with MGEs in the pan-genome, of which 44.7% (188 out of 421) were strain specific. These MGEs were mainly composed of gene families associated with plasmids (232), with a minor fraction of gene families associated with IS (90), prophage (93), and viruses (6) (Fig. 4d and e), many of them associated with “replication, recombination and repair” (Fisher’s exact test *P* value < 0.001) (Fig. S2a). Genes related to plasmid genes exhibited more strain specificity, whereas they were more prevalent in *A. salmonicida* ATCC 33658 and *A. aquatica* MX16A. Strains harboring a multitude of MGEs would readily mobilize genes within the genome or transmit them horizontally to other strains, which will contribute to the occurrence and directionality of the HGT process (45). The abundance of mobile elements, especially in the genome of *A. salmonicida* ATCC 33658, was likely connected to accelerated genome plasticity and gene transfer events. We found that 167 genes transferred by HGT (Table S4) might be attributed to MGEs, suggesting that MGEs play an important role in facilitating exchange, further emphasizing the important role of the MGE-mediated HGT in strains to their specific lifestyles. Generally, our results indicated that gene loss, HGTs, and MGEs seemed to be important evolutionary forces that contributed the genetic diversity in *Aeromonas* and facilitated rapid strain adaptation.

10.1128/mSystems.00252-19.8 TABLE S4 Predicted 167 horizontally transferred genes mediated by MGEs. Download Table S4, PDF file, 0.5 MB. Copyright © 2019 Zhong et al. 2019 Zhong et al. This content is distributed under the terms of the Creative Commons Attribution 4.0 International license.

### Gene patterns of virulence factors.

**FIG 5:**
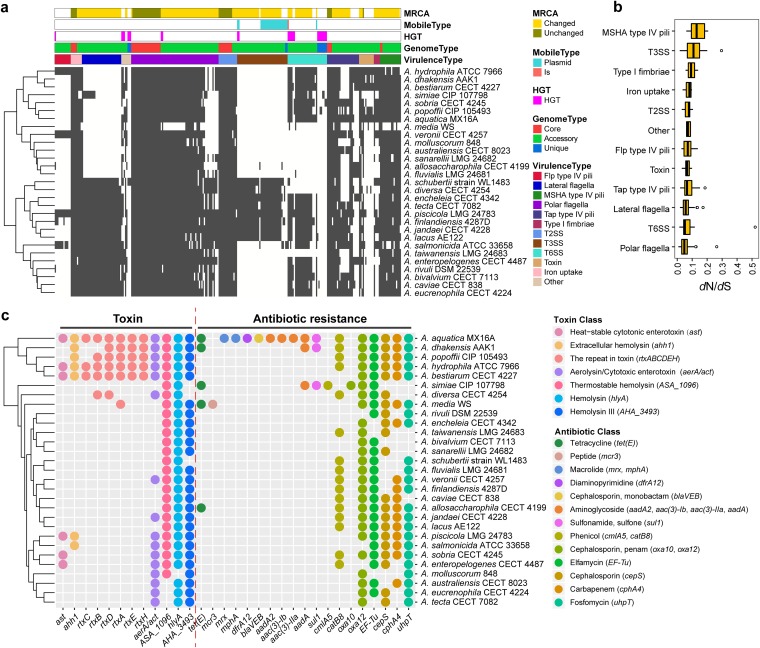
Distribution pattern of virulence factors in *Aeromonas* genomes. (a) Presence/absence of the virulence factors across the *Aeromonas* genomes. The tree on the left was clustered by calculating the Euclidean distance between species based on the presence or absence of the gene. Each column is a gene family of virulence factors, and each row is an *Aeromonas* strain. A gray bar indicates the presence of a gene. (b) Selection pressure of different types of virulence factors. (c) Presence of the toxin and antibiotic resistance genes. Each column represents a gene, and each circle represents presence of the gene. The color of the circle indicates the type of toxin or antibiotic resistance the gene encodes, and gene names are shown in parentheses after the toxin or antibiotic class.

The differential pathogenicity and infection of *Aeromonas* result from the presence or absence of potential virulence factors (48). To elucidate the relationship between virulence and the evolution of *Aeromonas*, we identified the virulence profiles of the 29 strains. We found 281 virulence-related genes in the pan-genome of *Aeromonas* involved in pili, iron uptake, flagella, secretion system, fimbriae, and toxin. Out of 281 genes, 46 genes were shared by all 29 strains (Table S3), while the remaining belonged to the variable genome. The widespread occurrence of these genes among *Aeromonas* strains emphasized their importance in the pathogenic mechanism. The appearance of polar flagella (24), T2SS (11), iron uptake (5), tap type IV pili (4), and mannose-sensitive hemagglutinin (MSHA) type IV pili (2) (Fig. 5a) in the core genome suggested common pathogenic mechanisms for their maintenance, irrespective of their source of isolation and not sufficient to determine virulence differences. Most of the virulence genes from these strains appeared to be inherited from the MRCA and preserved during speciation events. In addition, we observed a high variability in the virulence arsenal of *Aeromonas* species, in terms of number and composition, across the different genomes (e.g., the number of gene families per genome ranges from 86 to 249 [Table S5]). *Aeromonas* strains carrying diﬀerent virulence genes may have an important impact on their pathogenicity, and the presence of unique genes may be one of the factors affecting the ability to have different pathogenicity. In addition, large numbers of expansions and contractions of virulence gene families were evident in these bacterial genomes (Fig. 5a), which would enhance the evolutionary flexibility of these virulence genes. Although there are large changes in the size of gene families, the genes tended to undergo purifying selection instead of positive selection during evolution. We found that the single-copy virulence genes were subject to purifying selective pressure (Fig. 5b), which may be of major relevance for maintenance of pathogenicity, indicating that purifying selection is the force acting on the evolution of the single-copy virulence genes. In addition, constraint differences were also found in these different types of virulence factors (Kruskal-Wallis test *P* value < 0.001), where polar flagella appeared to be under stronger evolutionary constraints than other virulence types (*t* test *P* value < 0.001 [Fig. 5b]). These strains contained 26 MGEs carrying different virulence genes, indicating an important role of MGEs in the development and dissemination of virulence genes. In addition, there were 25 virulence genes potentially acquired by HGT, three of which were mediated by MGEs. Analysis of the gene locus associated with virulence showed that many virulence genes were enriched in physical clusters on genomes (Fig. S3b). For example, a physical cluster, *hut* cluster, involving in iron uptake, was shared by all 29 strains and experienced strong purifying selection. Also, there were still species-specific differences in the gene number and composition within some clusters. The clusters involved in T6SS and type I fimbriae were conserved in relatively greater numbers of strains.

10.1128/mSystems.00252-19.9 TABLE S5 Virulence factors were identified by type and the counts for each *Aeromonas* strain. The number of genes in each virulence type for each strain is displayed. Download Table S5, PDF file, 0.04 MB. Copyright © 2019 Zhong et al. 2019 Zhong et al. This content is distributed under the terms of the Creative Commons Attribution 4.0 International license.

Bacterial secretion systems are essential to bacterial pathogenicity, in which secretion systems T2SS, T3SS, and T6SS are critical for major virulence determinants for bacteria (49–51). T3SS was characterized as a virulence factor in *A. hydrophila* strains AH3 (52). In our results, T3SS-associated genes were mainly found in the genomes of *A. allosaccharophila* CECT 4199, *A. salmonicida* ATCC 33658, A. schubertii strain WL1483, A. diversa CECT 4254, A. encheleia CECT 4342, A. tecta CECT 7082, *A. piscicola* LMG 24783, A. finlandiensis 4287D, *A. jandaei* CECT 4228, and *A. lacus* AE122 (Fig. 5a and Table S5). In addition, T6SS was considered an important virulence mechanism for *A. hydrophila* SSU (53), and in our study, the T6SS was detected in 22 out of 29 strains. The results suggested that T3SS and T6SS were not essential to the pathogenicity of all *Aeromonas*, which was consistent with the results of a previous study (54). In contrast to T3SS and T6SS, we found that all 29 strains harbored T2SS genes, which indicates that T2SS is common for the pathogenicity of these strains.

In addition, diverse toxin genes cause the different toxicities of *Aeromonas* species (55). We observed the different distributions of the 12 genes encoding toxins across these genomes (Fig. 5c), which might contribute to the pathogenicity differences. The *A. aquatica* MX16A, *A. dhakensis* AAK1, *A. popoffii* CIP 105493, *A. hydrophila* ATCC 7966, and *A. bestiarum* CECT 4227 contained the most types of toxin-coding genes. The genes, such as *ASA_1096*, *hlyA*, and *AHA_3493*, can encode hemolysins causing apoptosis of the host cells (55), and these genes were detected in the vast majority of the strains, which indicated a common toxicity of hemolysin in these strains. We found that there were differences in the prevalence of the *ast*, *ahh1*, *aerA*/*act*, and *rtx* genes. The *A. aquatica* MX16A, *A. dhakensis* AAK1, *A. popoffii* CIP 105493, *A. hydrophila* ATCC 7966, *A. bestiarum* CECT 4227, *A. diversa* CECT 4254, and *A. media* WS possessed at least one *rtx* gene involved in RTX toxins. In addition, the cytotoxic-enterotoxin-encoding gene *ast* was shared by only six strains: *A. aquatica* MX16A, *A. hydrophila* ATCC 7966, A. bestiarum CECT 4227, *A. piscicola* LMG 24783, *A. sobria* CECT 4245, and *A. enteropelogenes* CECT 4487, while the gene *aerA*/*act* was present in 16 strains. The *aerA*/*act* gene encodes an aerolysin-related cytotoxic enterotoxin that is able to cause diarrheal diseases and wound infections (56, 57). The presence of this gene in these 16 strains indicated cytotoxic enterotoxin potential for these strains.

The pathogenic infection depends on the pathogenic potential of invading bacteria and bacterial ability to invade and evade host defenses, while the challenges of therapeutically resolving pathogenic infection come from antibiotic resistance (58). Antibiotic resistance in different degrees has been observed among *Aeromonas* species in clinical conditions (59, 60). We detected the distribution of resistance genes among the 29 strains and obtained 19 genes involved in a broad spectrum of antibiotic resistance, ranging from tetracycline antibiotic to fosfomycin antibiotic (Fig. 5c). These strains harbored different beta-lactam-encoding genes (*oxa10*, *cepS*, *blaVEB*, and *oxa12*) resistant to cephalosporins, which was in conformity with previous studies of variable resistance among *Aeromonas* with beta-lactamases being the major mechanism (3, 61). We observed that *A. aquatica* MX16A possessed 16 resistance genes and 7 of which were unique, which indicated that it possessed greater antibiotic resistance compared to other strains and warranted more investigations. We found that most strains harbored the genes resistant to chloramphenicol (*catB8*), cephalosporin (*oxa12*, *cepS*), penam (*oxa12*), elfamycin (elongation factor Tu [EF-Tu]), carbapenem (*cphA4*), and fosfomycin (*uhpT*), which have been reported (62, 63). The resistance of *Aeromonas* species to tetracycline and sulfonamides has been reported to be mainly caused by the *tet*(E) and *sul1* genes (64). In our study, we also detected five strains, *A. aquatica* MX16A, *A. dhakensis* AAK1, *A. simiae* CIP 107798, *A. media* WS, and *A. allosaccharophila* CECT 4199, that contained the tetracycline resistance gene *tet*(E), and three strains, *A. aquatica* MX16A, *A. dhakensis* AAK1, and *A. simiae* CIP 107798, contained sulfonamide resistance gene *sul1*. Moreover, genes such as *cepS*, *catB8*, *sul1*, *tet*(E), *oxa10*, *cmlA5*, *aac*(*3*)*-IIa*, *mphA*, *mrx*, *aac*(*3*)*-Ib*, *dfrA12*, and *aadA2* were detected to be introduced by plasmid-mediated HGT. Such prevalence of acquired antibiotic genes was consistent with previous studies that *Aeromonas* species would acquire antibiotic genes to adapt to environmental changes (65).

### Virulence patterns of *Aeromonas* in microbial communities.

Previous studies on virulence factors of *Aeromonas* focused mainly on isolated individual strains (26, 64), leaving their prevalence and importance in microbial communities understudied. Foods of animal origin are considered to play important roles in the transmission of *Aeromonas* to humans (17). Because the packaging is directly exposed to human beings, in order to understand the response of virulence factors of *Aeromonas* to diverse environments, we detected the dynamics of *Aeromonas* virulence factors in microbial communities.

**FIG 6:**
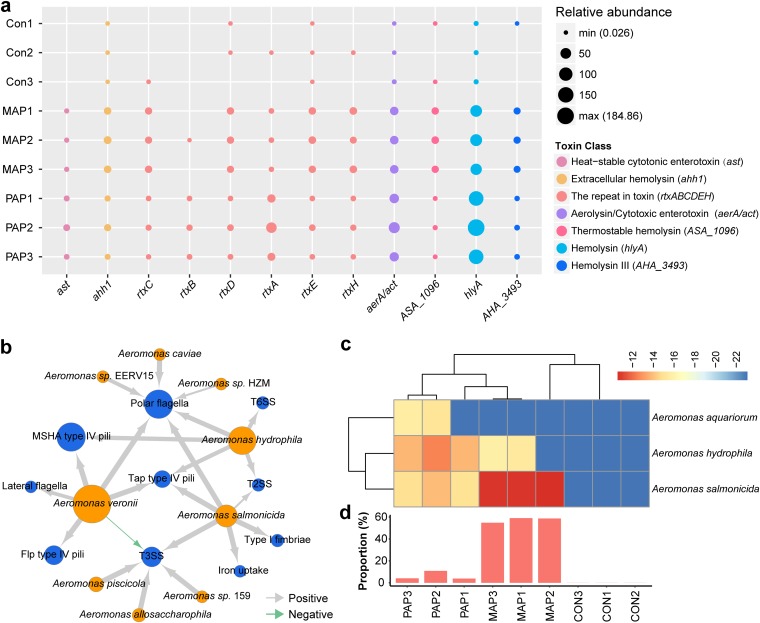
Diversity of virulence factors in microbial communities. (a) Distribution of toxin genes from the genus *Aeromonas* in different environmental conditions (packaging conditions). There were three groups, the control group (Con), modified-atmospheres packaging group (MAP), and penetrated-air packaging group (PAP), with three samples in each group. Each circle represents the relative abundance of genes measured by TPM. (b) Cooccurrence network between 10 types of virulence factors and 9 *Aeromonas* species. Blue nodes represent types of virulence factors, and orange nodes represent *Aeromonas* species. The size of the node (circle) represents the number of genes, while the thickness of the arrows represent the strength of the correlation. Gray arrows indicate positive correlation, and green arrows indicate negative correlation. (c) Heatmap plot showing the relative abundance of polymyxin resistance pathway of three *Aeromonas* species in microbial communities. (d) Contribution of *Aeromonas* genes to the polymyxin resistance pathway in microbial communities. Each bar displays the percentage of polymyxin resistance pathway of the genus *Aeromonas* to all strains. The *x* axis used in panel d applies to panel c also.

A recent study of microbial communities collected three groups of yellow-feather broilers packaged differently, the control group (CON) (stored 0 day), penetrated-air packaging group (PAP) (stored 4 days), and modified-atmospheres packaging group (MAP) (stored 8 days), with three samples in each group (66). The genus *Aeromonas* has been revealed to be abundant in these samples (66). Here, we detected 4,283 virulence genes in these nine samples, of which 1,266 were from the *Aeromonas* genus. Clear shifts of the abundance of virulence genes were observed after environmental changes. There were significantly lower levels of virulence genes in MAP-treated samples than in the other two groups (*t* test *P* value < 0.001) (Fig. S4a), while virulence genes from *Aeromonas* were present at a low abundance level in the control group than in the packaging groups (*t* test *P* value < 0.001) (Fig. S4b). After packaging, the types and relative abundance of virulence genes increased (Fig. S4c and S4d), especially those virulence genes from *Aeromonas*, whose relative abundance exceeded 49% of the total abundance (Fig. S4c). Such shifts of abundance and types of *Aeromonas* virulence genes indicated that packaging treatments lead to the increase of diversity of virulence during storage. Additionally, these virulence genes from *Aeromonas* were abundant in 10 virulence types, such as polar flagella, tap type IV pili, MSHA type IV pili, and T2SS (Fig. S4e), which shifted with abundance of *Aeromonas* (Fig. S4f). We also examined the different distribution of 12 toxin genes from *Aeromonas* in these samples, in which variation in toxin types would be the result of environmental changes (Fig. 6a). These results indicated that toxin genes of *Aeromonas* have differing patterns of cooccurrence over environment changes. The abundance of genes encoding hemolysin was higher than abundance of other toxins, which supported the universal retention of hemolysin pathogenicity in *Aeromonas*. The cooccurrence of virulence genes and *Aeromonas* species showed that Aeromonas veronii, Aeromonas hydrophila, and Aeromonas salmonicida carried genes involved in polar flagella and Tap type IV pili (Fig. 6b). In addition, we detected high abundance of polymyxin resistance pathways in these samples after different packaging, in which the high abundance of polymyxin resistance was mainly contributed by Aeromonas aquariorum, Aeromonas salmonicida, and Aeromonas hydrophila (Fig. 6c) and predominantly was originated from *Aeromonas* in MAP (57.5 ± 1.91%; Fig. 6d), indicating a possible acquisition of polymyxin resistance during environmental changes. The diversity of toxin genes in the microbial communities was also found from the MAP and PAP conditions. The virulence diversity of *Aeromonas* increased to the level matching these virulent *Aeromonas* species (such as *A. hydrophila*) when microbial communities respond to MAP and PAP.

10.1128/mSystems.00252-19.4 FIG S4 Distribution of virulence genes in microbial communities. (a) Comparison of relative abundance (measured by log_10_-transformed relative abundance) of virulence factors across samples. (b) Comparison of the relative abundance of virulence factors derived from the genus *Aeromonas*. (c) Taxonomic contribution of virulence genes to microbial communities at the genus level. (d) Virulence factors identified by type and the relative abundance for samples. Colored boxes represent virulence types. (e) Heatmap summarizing the relative abundances of virulence genes from the genus *Aeromonas* for each sample. (f) Comparison of the relative abundance between the 10 virulence types and the genus *Aeromonas* abundance. The 10 virulence types showed significant abundance correlations with the genus *Aeromonas*, with Pearson correlation *r *> 0.9 and *P < *0.01. Download FIG S4, TIF file, 2.5 MB. Copyright © 2019 Zhong et al. 2019 Zhong et al. This content is distributed under the terms of the Creative Commons Attribution 4.0 International license.

### Conclusions.

This study evaluated genetic diversity and evolutionary dynamics based on the pan-genome, elucidated virulence profiles in both single isolated species and microbial communities, and thereby provided a comprehensive understanding of the pathogenicity of *Aeromonas*.

The pan-genome of the 29 *Aeromonas* species was open, and newly sequenced strains contributed to an increasing number of genes for this genus. The pan-genome of *Aeromonas* harbored extensive genomic variability, about 84% of the pan-genome was variable, and the remaining 16% was constant and evolved under strong evolutionary constraints. By comparing the congruence between core genome phylogeny and pan-genome dendrogram, we observed that the two trees had significant congruence, which provided evidence that the core genome was an important factor influencing phylogeny. The evolutionary history of *Aeromonas* is marked not only by divergence among the core genes but also by the variable elements. Different selective pressures have operated on the diverse functions of *Aeromonas*, with the core genes and most accessory genes experiencing purifying selection. Four accessory genes have undergone positive selection, which may contribute to the impact of individual strains on functional variability. The large numbers of gene gains and losses indicated that *Aeromonas* genomes exhibit high levels of evolutionary plasticity, with a large number of gene families differing in size during evolution, which affected variable gene pools and facilitated rapid evolution. HGTs were drivers of genetic diversity that shaped *Aeromonas* genomes, as the existence of series genes related to IS, plasmid, prophage, and virus. Many of the genetic differences, especially for genes involved in virulence and resistance, were found within 29 *Aeromonas* species, with functions that mirror pathogenicity differences such as enterotoxin, hemolysin, and aerolysin.

Our study indicated that the 29 *Aeromonas* species present a pathogenic risk, as the virulence factors were prevalent. The selective constraints shaped virulence gene pools, and that acquisition of genes was important for specific virulence. The prevalence of hemolysin-encoding genes in these strains indicated that toxicity of hemolysin was ubiquitous in *Aeromonas*. In addition, *A. aquatica* MX16A, *A. dhakensis* AAK1, *A. popoffii* CIP 105493, *A. hydrophila* ATCC 7966, and *A. bestiarum* CECT 4227 were considered more pathogenic, as they contain more diverse types of genes related to toxicity. Notably, we also observed differences between the resistant genes, the HGT leading to specific resistance, suggesting that the abundance of some genes may reflect environmental selection. The acquired resistant genes indicated the greater antibiotic resistance of *A. aquatica* MX16A. Furthermore, we extended the scope of virulence to microbial communities, where we obtained a series of virulence genes for *Aeromonas*. The abundance of these genes varies under diverse environments to respond to the corresponding environment. The diversities of *Aeromonas* virulence in microbial communities increased to the level matching some of the most virulent *Aeromonas* species with the increased exposure to environments (such as in MAP and PAP conditions). The detection of virulence dynamics of *Aeromonas* over environment changes would facilitate the recognition of environmental conditions that trigger virulence increases. Hence, this study enhanced our knowledge of the diversity of *Aeromonas* virulence, from both single isolated species and microbial community settings, which will help to detect and prevent *Aeromonas* infection.

## MATERIALS AND METHODS

### Whole genomes of single isolated sequenced *Aeromonas*.

A data set comprising 29 genomes (draft and complete) from the genome database of the National Center for Biotechnology Information (NCBI) was obtained on 14 February 2017. The genome scale study used the most complete sampling of the diversity of the *Aeromonas* species thus far. We chose the genome with the highest level of completeness when several sequences were available for a given species. The sequences considered in the present study were from diverse sources, including humans, ﬁshes, and environments (see Table S1 in the supplemental material). These genomes used to reconstruct the pan-genome of *Aeromonas* were the most abundant species currently available in public databases.

### Ortholog identification.

Ortholog groups among the 29 genomes were determined using OrthoMCL (v2.0.9) (67) with default parameters: BLASTp E-value cutoff of 1e−5, percent match cutoff of 50, and MCL inflation index of 1.5. Homologous clusters were divided into core, accessory, and unique genomes. The core genome comprised shared genes within all of the genomes, while the accessory genome contained genes shared by at least 2 genomes but not all 29 genomes. The remaining genes in only one strain were strain-specific (unique) genes.

### Phylogeny analysis.

**(i) Pan-genome dendrogram analysis.** For the pan-genome dendrogram analysis, the distance matrix was calculated with orthologous relationships determined by OrthoMCL. The pairwise distance between each strain was calculated based on the presence and absence of orthologs using Manhattan distance. This Manhattan distance matrix was used as the phylogenetic distance and imported into MEGA (v5) (68). Finally, a pan-genome tree was constructed by the neighbor-joining method. Then the cutree function in R was employed on the tree. First, the number of groups was empirically determined in three groups (k = 3). We initially obtained three subtrees (n1 has 16 species, n2 has 3 species, and n3 has 10 species). Then, for the subtree (n1) with a species number greater than 10, we divided it into groups (k = 2) and obtained two subtrees (n1a has 9 species, n1b has 7 species). Finally, we obtained four main clusters containing 2 to 10 species each (clade I has 9 species, clade II has 7 species, clade III has 3 species, and clade IV has 10 species). These parameters are adjusted manually by referring to the previous studies (69, 70) to select the k value.

**(ii) Single-copy core gene phylogeny analysis.** For single-copy core gene phylogeny analysis, the single-copy core proteins from each genome were concatenated and subjected to multiple alignment using MUSCLE (v3.8.31) (71), and the regions that were divergent, misaligned, or with a large number of gaps were eliminated using the Gblocks (v0.91b) computer program (72) with the default parameter. Then a maximum likelihood tree was constructed based on the concatenated alignments using PHYLIP (v3.696) (73) with 100 bootstrap iterations. The tree was divided into groups using the cutree function in R, and the number of groups was determined empirically with four groups (k = 4) chosen; we preliminarily obtained the fourth subtree (n1 has 21 species, n2 has 6 species, n3 has 1 species, and n4 has 1 species). Then, for the subtree (n1) with the number of species greater than 10, we subdivided it into groups (k = 3) and obtained three subtrees (n1a has 7 species, n1b has 13 species, and n1c has 1 species), and then the n1b subtree was cut into two groups (k = 2). Finally, we obtained four main clusters containing 2 to 10 species each (clade 1 has 6 species, clade 2 has 7 species, clade 3 has 6 species, and clade 4 has 7 species). The congruence between single-copy core gene phylogeny and pan-genome dendrogram were computed by calculating normalized Robinson-Foulds (nRF) and normalized matching-cluster (nMC) scores using the ETE3 (74) and TreeCmp (36) computer programs, respectively. Consequently, higher nRF and nMC scores indicate a low level of congruence between two trees.

### Functional annotation.

The COG database was used for functional classification of the pan-genome. All genes were searched against the COG database using BLASTp with an E value of 1e−5, and the results were assigned to 26 functional categories. In addition, putative functions were identified using GO annotation within InterProScan (v.5) (75), and the enrichment of GO categories of proteins encoded by core genes in different categories was tested. The significant enrichments of COGs and GOs were measured by Fisher’s exact test.

### Evolutionary analysis.

The evolutionary pressure analyses were conducted on core gene sets using PAML (v 4.9a) (76). The rates of *dN* and *dS* were estimated using codeml program. To study expansion and contraction of gene families during the evolution of *Aeromonas*, a computational analysis of gene family sizes defines expansion or contraction of the gene families by comparing the cluster size of the ancestor to that of each of the current species was performed in CAFÉ (v3.1) (41) with a *P* value cutoff of 0.05. The single-copy tree was taken into account to infer the significance of change in gene family size in each branch.

### Identification of potentially horizontally transferred genes.

*Aeromonas* genomes were analyzed for recent transferred acquired genes using HGTector (v0.2.1) software (77). To select only recent acquisitions, each *Aeromonas* genome was searched individually using a minimum identity threshold of 90%, an E value of 1e−5, and 500 top-scoring matches.

### Annotation of mobile genetic elements.

To infer mobile elements, genes were aligned to plasmid sequences, insertion sequences, and phage sequences available in the NCBI RefSeq, ISfinder (78), and ACLAME (79) databaseS, respectively. Genes were determined as mobile elements at a 90% sequence identity threshold. In addition, these MGEs were used as inputs to HGTector AND then the genes were considered horizontally transferred genes mediated by MGEs if their best hit alignment had an identity greater than 90% and an E value below 1e−5.

### Identification of virulence factors and antibiotic resistance genes.

To identify the virulence factors of each species, genes were aligned against the Virulence Factors Database (VFDB) (80) using BLASTp. A gene was considered a potential virulence factor if its best hit alignment had an identity greater than 90% and an E value below 1e−10. In order to explore the profile of antibiotic resistance genes, gene alignments were performed on the Comprehensive Antibiotic Resistance Database (CARD) (81) using the same BLASTp parameters.

### Metagenomic data for microbial communities containing *Aeromonas* strains.

The metagenomes of chilled yellow-feathered broilers responding to modified-air packaging (MAP) and penetrated-air packaging (PAP) during storage and control samples with BioSamples identifier SAMN08123132 (BioProject accession no. PRJNA420874; SRA accession no. SRS2729591) were downloaded from NCBI.

### Assembly, annotation, and taxonomy analysis for metagenomic data.

The raw reads from each sample with a quality score lower than 20 were trimmed, and only reads longer than 50 were retained. The remaining reads were assembled using MEGAHIT (v1.1) (82), and the open reading frames (ORFs) were predicted using Prodigal (2.6.3) (83). Redundancy in the predicted ORF’s sequences were removed using Cd-hit (v4.6.6) (84). Afterwards, the nonredundant ORF sequences were searched against the VFDB database for identification of virulence-like ORFs using BLASTp with an E-value cutoff of 1e−10. A sequence was designated a virulence-like fragment if its best BLASTp alignment to virulent sequence showed a similarity of >90% and the alignment length was >25 amino acids. These virulence-like ORFs were compared with the NCBI NR database (ftp://ftp.ncbi.nlm.nih.gov/blast/db/) using diamond (85) program with an E value of 1e−5, and then the annotation of these ORFs was carried out using LCA algorithm analysis with MEGAN (v6.11.1). To compare the proportion of reads that mapped to a gene, TPM (transcripts per kilobase million) was calculated by the mapping reads to each ORF with Bowtie2 (v2.2.9) (86) and normalized for ORF length and then normalized for sequencing depth. TPM of each virulence genes was calculated and normalized in each sample to get the relative abundance of each gene.

### Data availability.

All results in this study are publicly available at the website (http://www.microbioinformatics.org/Aeromonas/).
