# Supplementary material for: Comprehensive Analysis Reveals the Evolution and Pathogenicity of Aeromonas, Viewed from Both Single Isolated Species and Microbial Communities
Source: mSystems. 2019 Oct 22;4(5):e00252-19. doi: 10.1128/mSystems.00252-19 (PMC6811364; doi:10.1128/mSystems.00252-19)
Supplement: TABLE S1 [file mSystems.00252-19-st001.pdf]

Supplementary Table S1. Bacterial strains and sources used for the genomic comparison of 29 *Aeromonas* species in this study.

Organism	RefSeq accession	Source	Size (Mb)	Genes	Proteins	Taxid
A. allosaccharophila CECT 4199	GCF_000819685.1	Diseased elver	4.66	4,325	4,065	656
A. aquatica MX16A	GCF_001895965.1	Environment, water	4.78	4,445	4,208	558964
A. australiensis CECT 8023	GCF_000819725.1	Environment, Irrigation water system	4.11	3,799	3,585	1114880
A. bestiarum CECT 4227	GCF_000819745.1	Diseased fish	4.69	4,236	4,075	105751
A. bivalvium CECT 7113	GCF_000819765.1	Cockles	4.3	4,002	3,765	440079
A. caviae CECT 838	GCF_000819785.1	Guinea pig	4.47	4,177	3,948	648
A. dhakensis AAK1	GCF_000315195.1	Human, Blood	4.76	4,373	4,207	1156800
A. diversa CECT 4254	GCF_000819805.1	Human, Wound	4.06	3,864	3,657	1268237
A. encheleia CECT 4342	GCF_000819825.1	Healthy eel in fresh water	4.47	4,145	3,970	73010
A. enteropelogenes CECT 4487	GCF_000819845.1	Human, Stool	4.47	4,158	3,958	29489
A. eucrenophila CECT 4224	GCF_000819865.1	Freshwater fish	4.54	4,159	3,996	649
A. finlandiensis 4287D	GCF_000764645.1	Environment, Lake water	4.72	4,475	4,074	1543375
A. fluvialis LMG 24681	GCF_000819885.1	Environmental, River water	3.9	3,686	3,408	591962
A. hydrophila ATCC 7966	GCF_000014805.1	Tin of milk with a fishy odour	4.74	4,283	4,118	380703
A. jandaei CECT 4228	GCF_000819955.1	Human, Stool	4.5	4,254	3,993	650
A. lacus AE122	GCF_000764665.1	Environment, Lake water	4.39	4,082	3,863	558884
A. media WS	GCF_000287215.2	Environment, Water	4.78	4,468	4,076	1208104
A. molluscorum 848	GCF_000388115.1	Wedge shells	4.24	4,090	3,596	1268236
A. piscicola LMG 24783	GCF_000820005.1	Salmon	5.18	4,917	4,595	600645
A. popoffii CIP 105493	GCF_000820025.1	Environmental, Water	4.76	4,472	4,126	70856
A. rivuli DSM 22539	GCF_000820045.1	Environment, Karst hardwater creek	4.53	4,292	4,040	648794
A. salmonicida ATCC 33658	GCF_001643305.1	Salmo salar	4.73	4,462	4,108	29491
A. sanarellii LMG 24682	GCF_000820085.1	Human, Wound	4.19	3,922	3,717	633415
A. schubertii strain WL1483	GCF_001447335.1	Snakehead Fish	4.4	4,268	3,854	652
A. simiae CIP 107798	GCF_000820125.1	Healthy monkey	3.99	3,815	3,551	218936
A. sobria CECT 4245	GCF_000820145.1	Fish	4.68	4,294	4,096	646
A. taiwanensis LMG 24683	GCF_000820165.1	Human, Wound	4.25	3,975	3,751	633417
A. tecta CECT 7082	GCF_000820185.1	Human, Stool	4.76	4,394	4,177	324617
A. veronii CECT 4257	GCF_000820225.1	Human, Sputum	4.52	4,179	3,999	197701
