# Supplementary material for: Comprehensive Analysis Reveals the Evolution and Pathogenicity of Aeromonas, Viewed from Both Single Isolated Species and Microbial Communities
Source: mSystems. 2019 Oct 22;4(5):e00252-19. doi: 10.1128/mSystems.00252-19 (PMC6811364; doi:10.1128/mSystems.00252-19)
Supplement: TABLE S2 [file mSystems.00252-19-st002.pdf]

Supplementary Table S2. Summary of homologous gene identified for 29 *Aeromonas* strains by OrthoMCL.

**Strain**	**Total gene families**	**Total gene counts**	**Core families**	**Core gene counts**	**Accessory families**	**Accessory gene counts**	**Unique families**	**Unique gene counts**
Total	10,144	113,003	1,645	48,085	4,825	61,132	3,674	3,786
A. hydrophila ATCC 7966	4,072	4,119	1,645	1,658	2,345	2,378	82	83
A. media WS	3,718	3,833	1,645	1,659	1,924	2,008	149	166
A. dhakensis AAK1	4,075	4,124	1,645	1,658	2,353	2,388	77	78
A. molluscorum 848	3,511	3,552	1,645	1,659	1,757	1,783	109	110
A. finlandiensis 4287D	3,981	4,025	1,645	1,660	2,216	2,244	120	121
A. lacus AE122	3,807	3,838	1,645	1,657	2,079	2,098	83	83
A. allosaccharophila CECT 4199	3,951	4,035	1,645	1,660	2,127	2,186	179	189
A. australiensis CECT 8023	3,543	3,572	1,645	1,655	1,775	1,793	123	124
A. bestiarum CECT 4227	3,984	4,023	1,645	1,657	2,260	2,286	79	80
A. bivalvium CECT 7113	3,654	3,694	1,645	1,657	1,932	1,959	77	78
A. caviae CECT 838	3,820	3,857	1,645	1,659	2,099	2,122	76	76
A. diversa CECT 4254	3,549	3,590	1,645	1,657	1,784	1,809	120	124
A. encheleia CECT 4342	3,873	3,913	1,645	1,661	2,142	2,165	86	87
A. enteropelogenes CECT 4487	3,858	3,895	1,645	1,658	2,084	2,107	129	130
A. eucrenophila CECT 4224	3,897	3,934	1,645	1,657	2,152	2,177	100	100
A. fluvialis LMG 24681	3,376	3,415	1,645	1,654	1,575	1,603	156	158
A. jandaei CECT 4228	3,902	3,948	1,645	1,658	2,176	2,209	81	81
A. piscicola LMG 24783	4,342	4,508	1,645	1,660	2,488	2,624	209	224
A. popoffii CIP 105493	3,962	4,071	1,645	1,657	2,080	2,173	237	241
A. rivuli DSM 22539	3,938	4,006	1,645	1,659	2,026	2,062	267	285
A. sanarellii LMG 24682	3,606	3,633	1,645	1,657	1,911	1,925	50	51
A. simiae CIP 107798	3,452	3,518	1,645	1,657	1,596	1,638	211	223
A. sobria CECT 4245	3,965	4,057	1,645	1,658	2,166	2,241	154	158
A. taiwanensis LMG 24683	3,650	3,684	1,645	1,661	1,932	1,950	73	73
A. tecta CECT 7082	4,082	4,121	1,645	1,659	2,326	2,351	111	111
A. veronii CECT 4257	3,906	3,955	1,645	1,663	2,184	2,215	77	77
A. schubertii strain WL1483	3,736	3,813	1,645	1,652	1,918	1,982	173	179
A. salmonicida ATCC 33658	3,996	4,061	1,645	1,657	2,177	2,222	174	182
A. aquatica MX16A	4,154	4,209	1,645	1,661	2,397	2,434	112	114
