# Supplementary figures and images for: Comprehensive Analysis Reveals the Evolution and Pathogenicity of Aeromonas, Viewed from Both Single Isolated Species and Microbial Communities
Source: mSystems. 2019 Oct 22;4(5):e00252-19. doi: 10.1128/mSystems.00252-19 (PMC6811364; doi:10.1128/mSystems.00252-19)

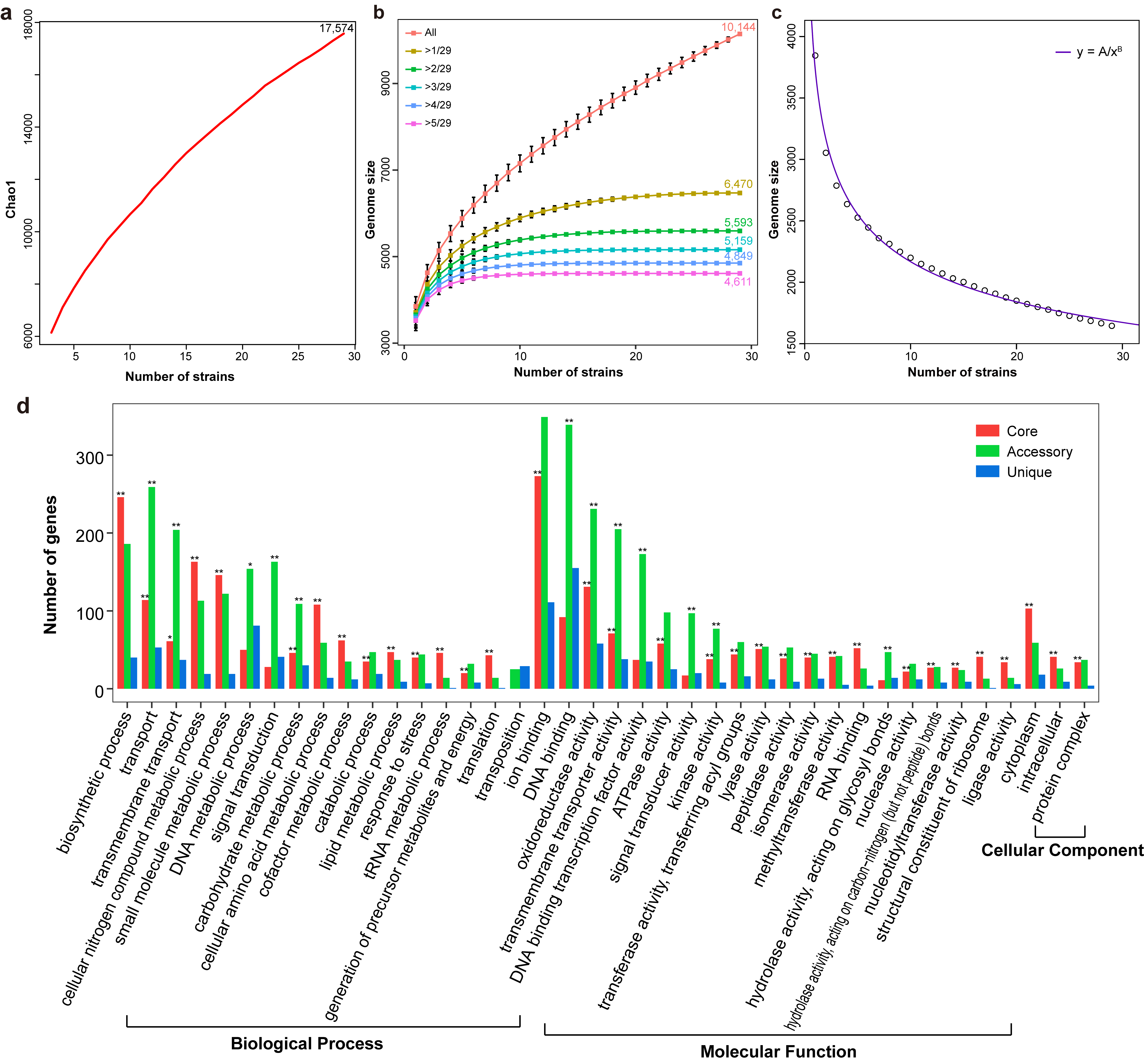

Supplement: FIG S1 [file mSystems.00252-19-sf001.tif]

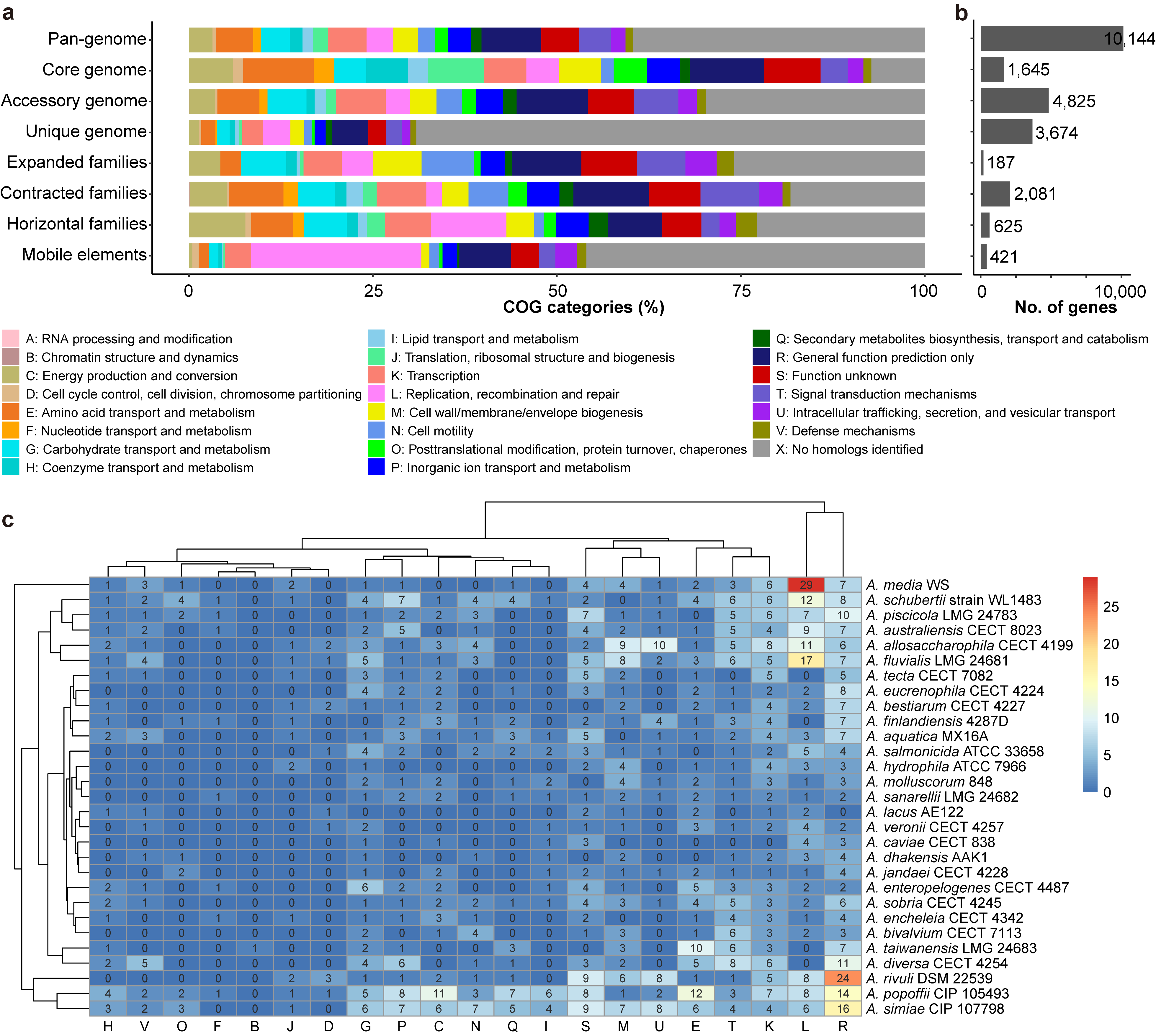

Supplement: FIG S2 [file mSystems.00252-19-sf002.tif]

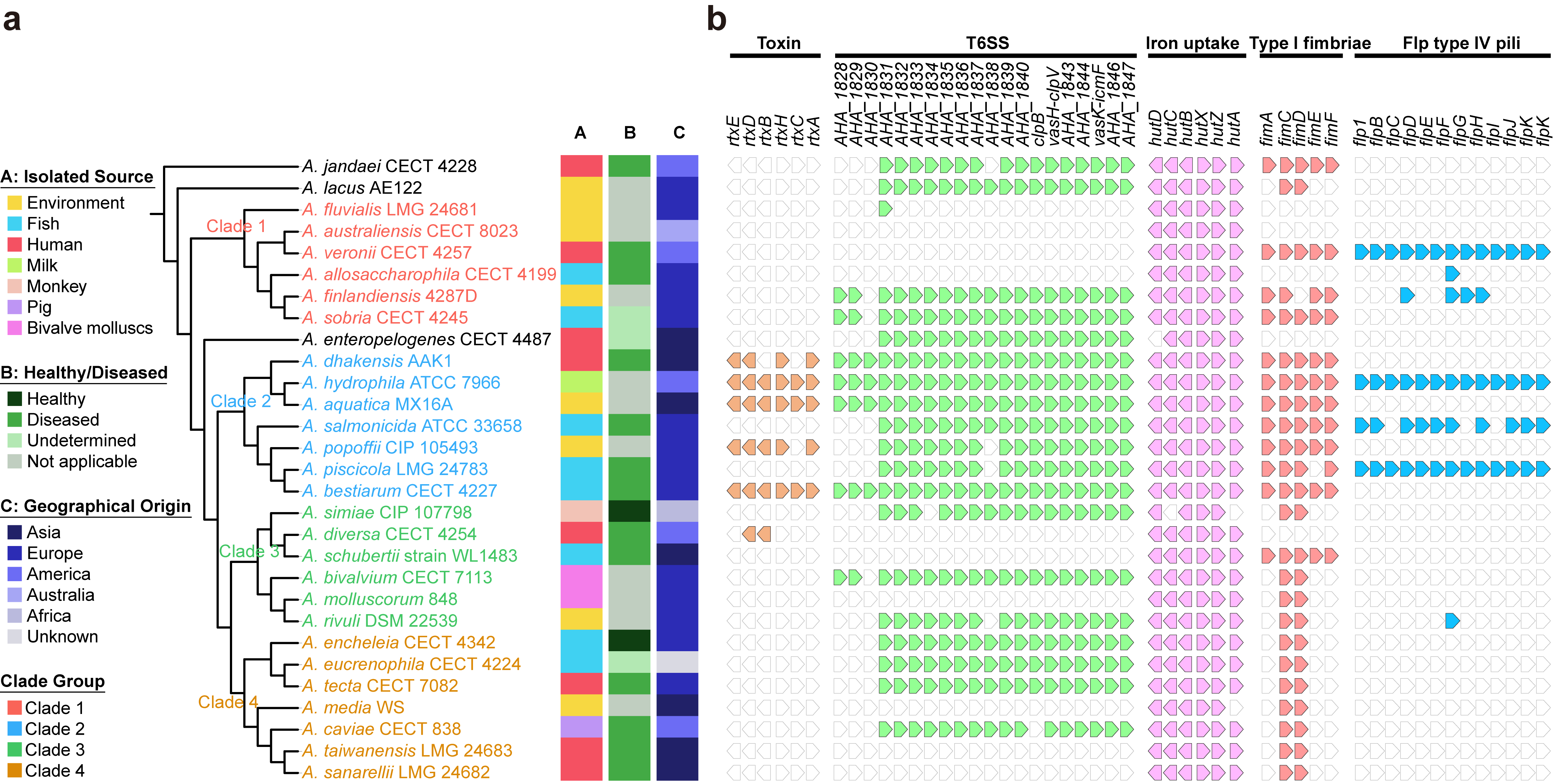

Supplement: FIG S3 [file mSystems.00252-19-sf003.tif]

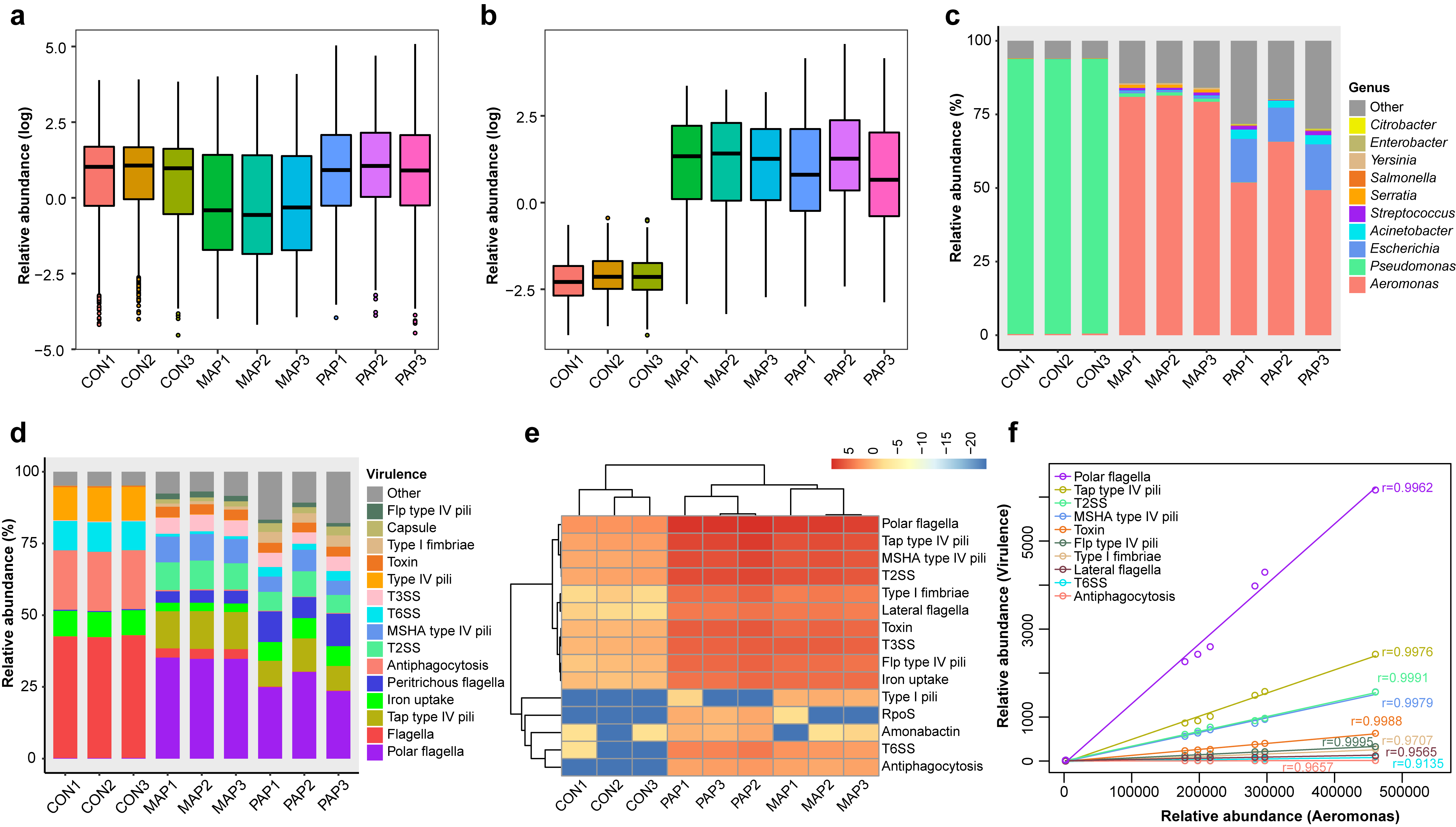

Supplement: FIG S4 [file mSystems.00252-19-sf004.tif]
