# Supplementary material for: Comprehensive Analysis Reveals the Evolution and Pathogenicity of Aeromonas, Viewed from Both Single Isolated Species and Microbial Communities
Source: mSystems. 2019 Oct 22;4(5):e00252-19. doi: 10.1128/mSystems.00252-19 (PMC6811364; doi:10.1128/mSystems.00252-19)
Supplement: TABLE S3 [file mSystems.00252-19-st003.pdf]

Supplementary Table S3. Summary of 16 predicted genus-specific genes of *Aeromonas* , 50 predicted horizontally transferred genes in core genome and 46 predicted virulence genes present in all 29 strains.

Gene family	Gene symbol	Genome type	Type	Category
Aeromonas_cluster634	*AHA_1282*	Core	Aeromonas-specific	Specific for the genus Aeromonas
Aeromonas_cluster20	*AHA_3819*	Core	Aeromonas-specific	Specific for the genus Aeromonas
Aeromonas_cluster956	*AHA_2198*	Core	Aeromonas-specific	Specific for the genus Aeromonas
Aeromonas_cluster717	*AHA_1552*	Core	Aeromonas-specific	Specific for the genus Aeromonas
Aeromonas_cluster160	*AHA_0150*	Core	Aeromonas-specific	Specific for the genus Aeromonas
Aeromonas_cluster693	*AHA_1510*	Core	Aeromonas-specific	Specific for the genus Aeromonas
Aeromonas_cluster354	*AHA_0627*	Core	Aeromonas-specific	Specific for the genus Aeromonas
Aeromonas_cluster311	*AHA_0510*	Core	Aeromonas-specific	Specific for the genus Aeromonas
Aeromonas_cluster281	*AHA_0437*	Core	Aeromonas-specific	Specific for the genus Aeromonas
Aeromonas_cluster534	*AHA_1006*	Core	Aeromonas-specific	Specific for the genus Aeromonas
Aeromonas_cluster690	*AHA_1499*	Core	Aeromonas-specific	Specific for the genus Aeromonas
Aeromonas_cluster1612	*AHA_4060*	Core	Aeromonas-specific	Specific for the genus Aeromonas
Aeromonas_cluster1324	*AHA_3339*	Core	Aeromonas-specific	Specific for the genus Aeromonas
Aeromonas_cluster356	*AHA_0638*	Core	Aeromonas-specific	Specific for the genus Aeromonas
Aeromonas_cluster1415	*AHA_3607*	Core	Aeromonas-specific	Specific for the genus Aeromonas
Aeromonas_cluster426	*AHA_0787*	Core	Aeromonas-specific	Specific for the genus Aeromonas
Aeromonas_cluster1058	*AHA_2472*	Core	HGT	Predicted horizontal transfer gene
Aeromonas_cluster1072	*AHA_2580*	Core	HGT	Predicted horizontal transfer gene
Aeromonas_cluster1084	*AHA_2616*	Core	HGT	Predicted horizontal transfer gene
Aeromonas_cluster1091	*AHA_2629*	Core	HGT	Predicted horizontal transfer gene
Aeromonas_cluster1096	*nth*	Core	HGT	Predicted horizontal transfer gene
Aeromonas_cluster111	*AHA_0046*	Core	HGT	Predicted horizontal transfer gene
Aeromonas_cluster1215	*crr*	Core	HGT	Predicted horizontal transfer gene
Aeromonas_cluster1231	*AHA_3118*	Core	HGT	Predicted horizontal transfer gene
Aeromonas_cluster1234	*gshB*	Core	HGT	Predicted horizontal transfer gene
Aeromonas_cluster1255	*rpe*	Core	HGT	Predicted horizontal transfer gene
Aeromonas_cluster1310	*AHA_3322*	Core	HGT	Predicted horizontal transfer gene
Aeromonas_cluster1313	*AHA_3325*	Core	HGT	Predicted horizontal transfer gene
Aeromonas_cluster1322	*glyA*	Core	HGT	Predicted horizontal transfer gene
Aeromonas_cluster1342	*mogA*	Core	HGT	Predicted horizontal transfer gene
Aeromonas_cluster1419	*gap-2*	Core	HGT	Predicted horizontal transfer gene
Aeromonas_cluster1457	*AHA_3698*	Core	HGT	Predicted horizontal transfer gene
Aeromonas_cluster147	*glyQ*	Core	HGT	Predicted horizontal transfer gene
Aeromonas_cluster1507	*AHA_3830*	Core	HGT	Predicted horizontal transfer gene
Aeromonas_cluster1551	*rpsI*	Core	HGT	Predicted horizontal transfer gene
Aeromonas_cluster1552	*rplM*	Core	HGT	Predicted horizontal transfer gene
Aeromonas_cluster1597	*rplA*	Core	HGT	Predicted horizontal transfer gene
Aeromonas_cluster1622	*pntB*	Core	HGT	Predicted horizontal transfer gene
Aeromonas_cluster1623	*pntA*	Core	HGT	Predicted horizontal transfer gene
Aeromonas_cluster1642	*potI*	Core	HGT	Predicted horizontal transfer gene
Aeromonas_cluster1654	*AHA_4189*	Core	HGT	Predicted horizontal transfer gene
Aeromonas_cluster1656	*AHA_4196*	Core	HGT	Predicted horizontal transfer gene
Aeromonas_cluster1677	*kbl*	Core	HGT	Predicted horizontal transfer gene
Aeromonas_cluster1678	*tdh*	Core	HGT	Predicted horizontal transfer gene
Aeromonas_cluster1693	*atpE*	Core	HGT	Predicted horizontal transfer gene
Aeromonas_cluster241	*rplD*	Core	HGT	Predicted horizontal transfer gene
Aeromonas_cluster246	*rpsC*	Core	HGT	Predicted horizontal transfer gene
Aeromonas_cluster249	*rpsQ*	Core	HGT	Predicted horizontal transfer gene
Aeromonas_cluster252	*rplE*	Core	HGT	Predicted horizontal transfer gene
Aeromonas_cluster314	*menB*	Core	HGT	Predicted horizontal transfer gene
Aeromonas_cluster32	*rfbB*	Core	HGT	Predicted horizontal transfer gene
Aeromonas_cluster341	*metJ*	Core	HGT	Predicted horizontal transfer gene
Aeromonas_cluster410	*xenB*	Core	HGT	Predicted horizontal transfer gene

Aeromonas_cluster411	*AHA_0738*	Core	HGT	Predicted horizontal transfer gene
Aeromonas_cluster412	*AHA_0739*	Core	HGT	Predicted horizontal transfer gene
Aeromonas_cluster501	*hfq*	Core	HGT	Predicted horizontal transfer gene
Aeromonas_cluster506	*rpmA*	Core	HGT	Predicted horizontal transfer gene
Aeromonas_cluster526	*crp*	Core	HGT	Predicted horizontal transfer gene
Aeromonas_cluster54	*zntA*	Core	HGT	Predicted horizontal transfer gene
Aeromonas_cluster664	*AHA_1404*	Core	HGT	Predicted horizontal transfer gene
Aeromonas_cluster732	*gmhA*	Core	HGT	Predicted horizontal transfer gene
Aeromonas_cluster794	*AHA_1732*	Core	HGT	Predicted horizontal transfer gene
Aeromonas_cluster813	*fdx*	Core	HGT	Predicted horizontal transfer gene
Aeromonas_cluster843	*AHA_1821*	Core	HGT	Predicted horizontal transfer gene
Aeromonas_cluster852	*lrp*	Core	HGT	Predicted horizontal transfer gene
Aeromonas_cluster890	*sucC*	Core	HGT	Predicted horizontal transfer gene
Aeromonas_cluster1135	*nueA*	Core	Polar flagella	Predicted virulence gene
Aeromonas_cluster1160	*fleR/flrC*	Core	Polar flagella	Predicted virulence gene
Aeromonas_cluster1161	*fleS/flrB*	Core	Polar flagella	Predicted virulence gene
Aeromonas_cluster1165	*flgK*	Core	Polar flagella	Predicted virulence gene
Aeromonas_cluster1166	*flgJ*	Core	Polar flagella	Predicted virulence gene
Aeromonas_cluster1167	*flgI*	Core	Polar flagella	Predicted virulence gene
Aeromonas_cluster1168	*flgH*	Core	Polar flagella	Predicted virulence gene
Aeromonas_cluster1169	*flgG*	Core	Polar flagella	Predicted virulence gene
Aeromonas_cluster1170	*flgF*	Core	Polar flagella	Predicted virulence gene
Aeromonas_cluster1171	*flgE*	Core	Polar flagella	Predicted virulence gene
Aeromonas_cluster1172	*flgD*	Core	Polar flagella	Predicted virulence gene
Aeromonas_cluster1173	*flgC*	Core	Polar flagella	Predicted virulence gene
Aeromonas_cluster1174	*flgB*	Core	Polar flagella	Predicted virulence gene
Aeromonas_cluster1175	*cheR-3*	Core	Polar flagella	Predicted virulence gene
Aeromonas_cluster1176	*cheV*	Core	Polar flagella	Predicted virulence gene
Aeromonas_cluster1177	*flgM*	Core	Polar flagella	Predicted virulence gene
Aeromonas_cluster1178	*flgN*	Core	Polar flagella	Predicted virulence gene
Aeromonas_cluster1259	*tapP*	Core	Tap type IV pili	Predicted virulence gene
Aeromonas_cluster1260	*tapO*	Core	Tap type IV pili	Predicted virulence gene
Aeromonas_cluster1261	*tapM*	Core	Tap type IV pili	Predicted virulence gene
Aeromonas_cluster329	*exeC*	Core	T2SS	Predicted virulence gene
Aeromonas_cluster330	*exeD*	Core	T2SS	Predicted virulence gene
Aeromonas_cluster331	*exeE*	Core	T2SS	Predicted virulence gene
Aeromonas_cluster332	*exeF*	Core	T2SS	Predicted virulence gene
Aeromonas_cluster333	*exeG*	Core	T2SS	Predicted virulence gene
Aeromonas_cluster334	*exeH*	Core	T2SS	Predicted virulence gene
Aeromonas_cluster335	*exeI*	Core	T2SS	Predicted virulence gene
Aeromonas_cluster336	*exeJ*	Core	T2SS	Predicted virulence gene
Aeromonas_cluster337	*exeK*	Core	T2SS	Predicted virulence gene
Aeromonas_cluster338	*exeM*	Core	T2SS	Predicted virulence gene
Aeromonas_cluster339	*exeN*	Core	T2SS	Predicted virulence gene
Aeromonas_cluster367	*motX*	Core	Polar flagella	Predicted virulence gene
Aeromonas_cluster519	*hutB*	Core	Iron uptake	Predicted virulence gene
Aeromonas_cluster520	*hutX*	Core	Iron uptake	Predicted virulence gene
Aeromonas_cluster521	*hutZ*	Core	Iron uptake	Predicted virulence gene
Aeromonas_cluster522	*ASA_3331*	Core	Iron uptake	Predicted virulence gene
Aeromonas_cluster523	*ASA_3330*	Core	Iron uptake	Predicted virulence gene
Aeromonas_cluster62	*mshJ*	Core	MSHA type IV pili	Predicted virulence gene
Aeromonas_cluster63	*mshI*	Core	MSHA type IV pili	Predicted virulence gene
Aeromonas_cluster653	*cheB-2*	Core	Polar flagella	Predicted virulence gene
Aeromonas_cluster654	*pomB*	Core	Polar flagella	Predicted virulence gene
Aeromonas_cluster655	*B565_1123*	Core	Polar flagella	Predicted virulence gene
Aeromonas_cluster656	*cheW*	Core	Polar flagella	Predicted virulence gene
Aeromonas_cluster782	*flaG*	Core	Polar flagella	Predicted virulence gene
Aeromonas_cluster7	*flaB*	Core	Polar flagella	Predicted virulence gene

Aeromonas_cluster818	*tapF*	Core	Tap type IV pili	Predicted virulence gene
