# Supplementary material for: Comprehensive Analysis Reveals the Evolution and Pathogenicity of Aeromonas, Viewed from Both Single Isolated Species and Microbial Communities
Source: mSystems. 2019 Oct 22;4(5):e00252-19. doi: 10.1128/mSystems.00252-19 (PMC6811364; doi:10.1128/mSystems.00252-19)
Supplement: TABLE S4 [file mSystems.00252-19-st004.pdf]

Supplementary Table S4. Predicted 167 horizontally transferred genes mediated by MGEs.

Gene symbol	Mobile Element	Genome Type	Strain
BN1125_RS13630	Insertion sequence	Accessory	*A. allosaccharophila* CECT 4199
BN1125_RS09275	Insertion sequence	Accessory	*A. allosaccharophila* CECT 4199
BN1115_RS19170	Insertion sequence	Accessory	*A. allosaccharophila* CECT 4199
BN1125_RS13625	Insertion sequence	Accessory	*A. allosaccharophila* CECT 4199, *A. fluvialis* LMG 24681, *A. rivuli* DSM 22539, *A. simiae* CIP 107798
BN1115_RS09810	Insertion sequence	Accessory	*A. allosaccharophila* CECT 4199, *A. popoffii* CIP 105493, *A. salmonicida* ATCC 33658
flhA	Insertion sequence	Accessory	*A. australiensis* CECT 8023, *A. popoffii* CIP 105493
BN1119_RS17070	Insertion sequence	Accessory	*A. bivalvium* CECT 7113, *A. encheleia* CECT 4342, *A. enteropelogenes* CECT 4487, *A. piscicola* LMG 24783, *A. popoffii* CIP 105493, *A. rivuli* DSM 22539, *A. simiae* CIP 107798, *A. sobria* CECT 4245, *A. veronii* CECT 4257, *A. schubertii* strain WL1483
BN1119_RS00140	Insertion sequence	Accessory	*A. bivalvium* CECT 7113, *A. encheleia* CECT 4342, *A. enteropelogenes* CECT 4487, *A. piscicola* LMG 24783, *A. popoffii* CIP 105493, *A. simiae* CIP 107798, *A. sobria* CECT 4245, *A. veronii* CECT 4257, *A. schubertii* strain WL1483
AQU_RS21120	Insertion sequence	Accessory	*A. dhakensis* AAK1, *A. allosaccharophila* CECT 4199, *A. bestiarum* CECT 4227, *A. bivalvium* CECT 7113, *A. caviae* CECT 838, *A. diversa* CECT 4254, *A. jandaei* CECT 4228, *A. sanarellii* LMG 24682, *A. sobria* CECT 4245, *A. veronii* CECT 4257, *A. schubertii* strain WL1483, *A. salmonicida* ATCC 33658
BN1125_RS05635	Insertion sequence	Accessory	*A. dhakensis* AAK1, *A. allosaccharophila* CECT 4199, *A. bivalvium* CECT 7113, *A. enteropelogenes* CECT 4487, *A. sobria* CECT 4245, *A. taiwanensis* LMG 24683, *A. veronii* CECT 4257
LD28_RS0121450	Insertion sequence	Accessory	*A. finlandiensis* 4287D
BN1125_RS07670	Insertion sequence	Accessory	*A. media* WS
BN1125_RS10465	Insertion sequence	Accessory	*A. media* WS
B224_RS07790	Insertion sequence	Accessory	*A. media* WS, *A. allosaccharophila* CECT 4199, *A. australiensis* CECT 8023, *A. piscicola* LMG 24783
B224_RS17020	Insertion sequence	Accessory	*A. media* WS, *A. allosaccharophila* CECT 4199, *A. australiensis* CECT 8023, *A. schubertii* strain WL1483
B224_RS01930	Insertion sequence	Accessory	*A. media* WS, *A. australiensis* CECT 8023
B224_RS00725	Insertion sequence	Accessory	*A. media* WS, *A. australiensis* CECT 8023, *A. popoffii* CIP 105493
B224_RS01095	Insertion sequence	Accessory	*A. media* WS, *A. diversa* CECT 4254, *A. schubertii* strain WL1483
BN1125_RS06775	Insertion sequence	Accessory	*A. media* WS, *A. fluvialis* LMG 24681, *A. popoffii* CIP 105493, *A. simiae* CIP 107798
G113_RS12770	Insertion sequence	Accessory	*A. molluscorum* 848, *A. bestiarum* CECT 4227, *A. bivalvium* CECT 7113, *A. simiae* CIP 107798, *A. taiwanensis* LMG 24683
AHA_1119	Insertion sequence	Accessory	*A. schubertii* strain WL1483
WL1483_RS00020	Insertion sequence	Accessory	*A. schubertii* strain WL1483
BN1125_RS13620	Insertion sequence	Accessory	*A. simiae* CIP 107798

ampC	Plasmid	Accessory	*A. allosaccharophila* CECT 4199, *A. bestiarum* CECT 4227, *A. piscicola* LMG 24783, *A. sanarellii* LMG 24682
BOQ57_RS11335	Plasmid	Accessory	*A. aquatica* MX16A
BN1117_RS12680	Plasmid	Accessory	*A. australiensis* CECT 8023
BN1117_RS08530	Plasmid	Accessory	*A. australiensis* CECT 8023, *A. bivalvium* CECT 7113
BN1117_RS12685	Plasmid	Accessory	*A. australiensis* CECT 8023, *A. jandaei* CECT 4228
BN1117_RS03145	Plasmid	Accessory	*A. australiensis* CECT 8023, *A. salmonicida* ATCC 33658
AHA_0625	Plasmid	Accessory	*A. bivalvium* CECT 7113
BN1119_RS14860	Plasmid	Accessory	*A. bivalvium* CECT 7113
BN1119_RS11040	Plasmid	Accessory	*A. bivalvium* CECT 7113
AHA_0214	Plasmid	Accessory	*A. bivalvium* CECT 7113, *A. caviae* CECT 838, *A. eucrenophila* CECT 4224, *A. sanarellii* LMG 24682, *A. taiwanensis* LMG 24683
BN1119_RS17945	Plasmid	Accessory	*A. bivalvium* CECT 7113, *A. salmonicida* ATCC 33658
AHA_0037	Plasmid	Accessory	*A. dhakensis* AAK1
AQU_RS18805	Plasmid	Accessory	*A. dhakensis* AAK1, *A. allosaccharophila* CECT 4199, *A. simiae* CIP 107798, *A. aquatica* MX16A
AQU_RS18795	Plasmid	Accessory	*A. dhakensis* AAK1, *A. aquatica* MX16A
AQU_RS18815	Plasmid	Accessory	*A. dhakensis* AAK1, *A. molluscorum* 848, *A. aquatica* MX16A
AQU_RS18845	Plasmid	Accessory	*A. dhakensis* AAK1, *A. simiae* CIP 107798, *A. aquatica* MX16A
AQU_RS18825	Plasmid	Accessory	*A. dhakensis* AAK1, *A. simiae* CIP 107798, *A. aquatica* MX16A
AQU_RS18840	Plasmid	Accessory	*A. dhakensis* AAK1, *A. simiae* CIP 107798, *A. aquatica* MX16A
AQU_RS18810	Plasmid	Accessory	*A. dhakensis* AAK1, *A. simiae* CIP 107798, *A. aquatica* MX16A
AHA_2243	Plasmid	Accessory	*A. finlandiensis* 4287D, *A. allosaccharophila* CECT 4199, *A. piscicola* LMG 24783, *A. sobria* CECT 4245, *A. veronii* CECT 4257, *A. salmonicida* ATCC 33658
AHA_3133	Plasmid	Accessory	*A. finlandiensis* 4287D, *A. lacus* AE122, *A. allosaccharophila* CECT 4199, *A. encheleia* CECT 4342, *A. jandaei* CECT 4228, *A. piscicola* LMG 24783, *A. sobria* CECT 4245, *A. veronii* CECT 4257, *A. salmonicida* ATCC 33658
fliI	Plasmid	Accessory	*A. finlandiensis* 4287D, *A. lacus* AE122, *A. diversa* CECT 4254, *A. encheleia* CECT 4342, *A. jandaei* CECT 4228, *A. piscicola* LMG 24783, *A. tecta* CECT 7082, *A. schubertii* strain WL1483
B224_RS04160	Plasmid	Accessory	*A. media* WS, *A. allosaccharophila* CECT 4199, *A. bivalvium* CECT 7113, *A. schubertii* strain WL1483, *A. aquatica* MX16A
B224_RS17435	Plasmid	Accessory	*A. media* WS, *A. allosaccharophila* CECT 4199, *A. fluvialis* LMG 24681, *A. aquatica* MX16A
B224_RS06355	Plasmid	Accessory	*A. media* WS, *A. aquatica* MX16A
AHA_3130	Plasmid	Accessory	*A. media* WS, *A. caviae* CECT 838, *A. encheleia* CECT 4342, *A. eucrenophila* CECT 4224, *A. sanarellii* LMG 24682, *A. taiwanensis* LMG 24683

B224_RS11130	Plasmid	Accessory	*A. media* WS, *A. dhakensis* AAK1, *A. allosaccharophila* CECT 4199, *A. simiae* CIP 107798, *A. aquatica* MX16A
B224_RS04110	Plasmid	Accessory	*A. media* WS, *A. dhakensis* AAK1, *A. molluscorum* 848, *A. encheleia* CECT 4342, *A. rivuli* DSM 22539, *A. aquatica* MX16A
B224_RS11985	Plasmid	Accessory	*A. media* WS, *A. simiae* CIP 107798, *A. salmonicida* ATCC 33658, *A. aquatica* MX16A
G113_RS12775	Plasmid	Accessory	*A. molluscorum* 848, *A. bestiarum* CECT 4227, *A. bivalvium* CECT 7113, *A. caviae* CECT 838, *A. simiae* CIP 107798, *A. taiwanensis* LMG 24683, *A. aquatica* MX16A
BN1128_RS22125	Plasmid	Accessory	*A. piscicola* LMG 24783
BN1128_RS22830	Plasmid	Accessory	*A. piscicola* LMG 24783, *A. rivuli* DSM 22539, *A. simiae* CIP 107798, *A. salmonicida* ATCC 33658, *A. aquatica* MX16A
AHA_4095	Plasmid	Accessory	*A. rivuli* DSM 22539
AXW79_RS08025	Plasmid	Accessory	*A. salmonicida* ATCC 33658
AXW79_RS18750	Plasmid	Accessory	*A. salmonicida* ATCC 33658
AXW79_RS18755	Plasmid	Accessory	*A. salmonicida* ATCC 33658
BN1134_RS03915	Plasmid	Accessory	*A. simiae* CIP 107798
BN1134_RS03975	Plasmid	Accessory	*A. simiae* CIP 107798
BN1134_RS03955	Plasmid	Accessory	*A. simiae* CIP 107798
BN1134_RS04020	Plasmid	Accessory	*A. simiae* CIP 107798
BN1134_RS04005	Plasmid	Accessory	*A. simiae* CIP 107798, *A. aquatica* MX16A
BN1134_RS04025	Plasmid	Accessory	*A. simiae* CIP 107798, *A. aquatica* MX16A
BN1120_RS15340	Prophage	Accessory	*A. caviae* CECT 838
AQU_RS20380	Prophage	Accessory	*A. dhakensis* AAK1
AQU_RS20290	Prophage	Accessory	*A. dhakensis* AAK1, *A. encheleia* CECT 4342, *A. piscicola* LMG 24783
AQU_RS20365	Prophage	Accessory	*A. dhakensis* AAK1, *A. eucrenophila* CECT 4224
AQU_RS20325	Prophage	Accessory	*A. dhakensis* AAK1, *A. molluscorum* 848, *A. encheleia* CECT 4342, *A. enteropelogenes* CECT 4487, *A. eucrenophila* CECT 4224, *A. piscicola* LMG 24783
AQU_RS20305	Prophage	Accessory	*A. dhakensis* AAK1, *A. molluscorum* 848, *A. encheleia* CECT 4342, *A. enteropelogenes* CECT 4487, *A. eucrenophila* CECT 4224, *A. piscicola* LMG 24783
AQU_RS20320	Prophage	Accessory	*A. dhakensis* AAK1, *A. molluscorum* 848, *A. enteropelogenes* CECT 4487, *A. eucrenophila* CECT 4224, *A. piscicola* LMG 24783
BN1122_RS18270	Prophage	Accessory	*A. encheleia* CECT 4342, *A. enteropelogenes* CECT 4487
BN1122_RS18200	Prophage	Accessory	*A. encheleia* CECT 4342, *A. eucrenophila* CECT 4224, *A. piscicola* LMG 24783
BN1122_RS18195	Prophage	Accessory	*A. encheleia* CECT 4342, *A. piscicola* LMG 24783
BN1124_RS14960	Prophage	Accessory	*A. eucrenophila* CECT 4224
BN1124_RS14845	Prophage	Accessory	*A. eucrenophila* CECT 4224
AHA_2066	Prophage	Accessory	*A. eucrenophila* CECT 4224
BN1124_RS14770	Prophage	Accessory	*A. eucrenophila* CECT 4224, *A. piscicola* LMG 24783
AHA_1073	Prophage	Accessory	*A. hydrophila* ATCC 7966, *A. dhakensis* AAK1, *A. fluvialis* LMG 24681, *A. sobria* CECT 4245, *A. taiwanensis* LMG 24683

AHA_2061	Prophage	Accessory	*A. hydrophila* ATCC 7966, *A. encheleia* CECT 4342, *A. enteropelogenes* CECT 4487, *A. piscicola* LMG 24783, *A. schubertii* strain WL1483, *A. salmonicida* ATCC 33658
BN1128_RS18880	Prophage	Accessory	*A. piscicola* LMG 24783
BN1128_RS18910	Prophage	Accessory	*A. piscicola* LMG 24783
BN1135_RS12830	Prophage	Accessory	*A. sobria* CECT 4245, *A. taiwanensis* LMG 24683
AQU_RS20240	Virus	Accessory	*A. dhakensis* AAK1, *A. encheleia* CECT 4342, *A. eucrenophila* CECT 4224, *A. piscicola* LMG 24783
G113_RS02010	Virus	Accessory	*A. molluscorum* 848
zntA	Plasmid	core	*A. molluscorum* 848
BOQ57_RS11435	Insertion sequence	Unique	*A. aquatica* MX16A
BN1117_RS08205	Insertion sequence	Unique	*A. australiensis* CECT 8023
BN1117_RS18085	Insertion sequence	Unique	*A. australiensis* CECT 8023
BN1125_RS00280	Insertion sequence	Unique	*A. fluvialis* LMG 24681
B224_RS06475	Insertion sequence	Unique	*A. media* WS
B224_RS08155	Insertion sequence	Unique	*A. media* WS
B224_RS00325	Insertion sequence	Unique	*A. media* WS
B224_RS09735	Insertion sequence	Unique	*A. media* WS
B224_RS04085	Insertion sequence	Unique	*A. media* WS
B224_RS09740	Insertion sequence	Unique	*A. media* WS
BN1128_RS21995	Insertion sequence	Unique	*A. piscicola* LMG 24783
BN1128_RS06465	Insertion sequence	Unique	*A. piscicola* LMG 24783
BN1129_RS00195	Insertion sequence	Unique	*A. popoffii* CIP 105493
BN1129_RS04455	Insertion sequence	Unique	*A. popoffii* CIP 105493
WL1483_RS12635	Insertion sequence	Unique	*A. schubertii* strain WL1483
WL1483_RS02235	Insertion sequence	Unique	*A. schubertii* strain WL1483
WL1483_RS12545	Insertion sequence	Unique	*A. schubertii* strain WL1483
WL1483_RS16190	Insertion sequence	Unique	*A. schubertii* strain WL1483
WL1483_RS00170	Insertion sequence	Unique	*A. schubertii* strain WL1483
BN1134_RS17400	Insertion sequence	Unique	*A. simiae* CIP 107798
BN1134_RS03920	Insertion sequence	Unique	*A. simiae* CIP 107798
BN1115_RS13655	Plasmid	Unique	*A. allosaccharophila* CECT 4199
BN1115_RS00095	Plasmid	Unique	*A. allosaccharophila* CECT 4199
BOQ57_RS11275	Plasmid	Unique	*A. aquatica* MX16A
BOQ57_RS11515	Plasmid	Unique	*A. aquatica* MX16A
BOQ57_RS11375	Plasmid	Unique	*A. aquatica* MX16A
BOQ57_RS11420	Plasmid	Unique	*A. aquatica* MX16A
BOQ57_RS11425	Plasmid	Unique	*A. aquatica* MX16A
BOQ57_RS11350	Plasmid	Unique	*A. aquatica* MX16A
BOQ57_RS11530	Plasmid	Unique	*A. aquatica* MX16A
BOQ57_RS11510	Plasmid	Unique	*A. aquatica* MX16A
BOQ57_RS11535	Plasmid	Unique	*A. aquatica* MX16A
BOQ57_RS11440	Plasmid	Unique	*A. aquatica* MX16A
BOQ57_RS11450	Plasmid	Unique	*A. aquatica* MX16A
BOQ57_RS11355	Plasmid	Unique	*A. aquatica* MX16A
BOQ57_RS11445	Plasmid	Unique	*A. aquatica* MX16A
BOQ57_RS11525	Plasmid	Unique	*A. aquatica* MX16A
BOQ57_RS11390	Plasmid	Unique	*A. aquatica* MX16A
BOQ57_RS11200	Plasmid	Unique	*A. aquatica* MX16A
BOQ57_RS11380	Plasmid	Unique	*A. aquatica* MX16A
BOQ57_RS11345	Plasmid	Unique	*A. aquatica* MX16A
BOQ57_RS11520	Plasmid	Unique	*A. aquatica* MX16A
BOQ57_RS11540	Plasmid	Unique	*A. aquatica* MX16A
BOQ57_RS11285	Plasmid	Unique	*A. aquatica* MX16A
BOQ57_RS11270	Plasmid	Unique	*A. aquatica* MX16A
BOQ57_RS11385	Plasmid	Unique	*A. aquatica* MX16A

BOQ57_RS11410	Plasmid	Unique	*A. aquatica* MX16A
BOQ57_RS11455	Plasmid	Unique	*A. aquatica* MX16A
BOQ57_RS11545	Plasmid	Unique	*A. aquatica* MX16A
BOQ57_RS11415	Plasmid	Unique	*A. aquatica* MX16A
BOQ57_RS11290	Plasmid	Unique	*A. aquatica* MX16A
BOQ57_RS11240	Plasmid	Unique	*A. aquatica* MX16A
BOQ57_RS11235	Plasmid	Unique	*A. aquatica* MX16A
BOQ57_RS11340	Plasmid	Unique	*A. aquatica* MX16A
BN1117_RS03960	Plasmid	Unique	*A. australiensis* CECT 8023
BN1118_RS09595	Plasmid	Unique	*A. bestiarum* CECT 4227
BN1121_RS14320	Plasmid	Unique	*A. diversa* CECT 4254
BN1121_RS14315	Plasmid	Unique	*A. diversa* CECT 4254
BN1121_RS02805	Plasmid	Unique	*A. diversa* CECT 4254
B224_RS04125	Plasmid	Unique	*A. media* WS
B224_RS04115	Plasmid	Unique	*A. media* WS
B224_RS04120	Plasmid	Unique	*A. media* WS
B224_RS04140	Plasmid	Unique	*A. media* WS
B224_RS04130	Plasmid	Unique	*A. media* WS
B224_RS21885	Plasmid	Unique	*A. media* WS
B224_RS06455	Plasmid	Unique	*A. media* WS
B224_RS06440	Plasmid	Unique	*A. media* WS
B224_RS04135	Plasmid	Unique	*A. media* WS
BN1129_RS11635	Plasmid	Unique	*A. popoffii* CIP 105493
BN1130_RS17465	Plasmid	Unique	*A. rivuli* DSM 22539
AXW79_RS09240	Plasmid	Unique	*A. salmonicida* ATCC 33658
AXW79_RS09250	Plasmid	Unique	*A. salmonicida* ATCC 33658
BN1134_RS03990	Plasmid	Unique	*A. simiae* CIP 107798
BN1134_RS03995	Plasmid	Unique	*A. simiae* CIP 107798
BN1134_RS03980	Plasmid	Unique	*A. simiae* CIP 107798
BN1134_RS14735	Plasmid	Unique	*A. simiae* CIP 107798
BN1118_RS19665	Prophage	Unique	*A. bestiarum* CECT 4227
BN1130_RS19510	Virus	Unique	*A. rivuli* DSM 22539
BN1130_RS19475	Virus	Unique	*A. rivuli* DSM 22539
BN1130_RS19485	Virus	Unique	*A. rivuli* DSM 22539
