# Supplementary material for: Comprehensive Analysis Reveals the Evolution and Pathogenicity of Aeromonas, Viewed from Both Single Isolated Species and Microbial Communities
Source: mSystems. 2019 Oct 22;4(5):e00252-19. doi: 10.1128/mSystems.00252-19 (PMC6811364; doi:10.1128/mSystems.00252-19)
Supplement: TABLE S5 [file mSystems.00252-19-st005.pdf]

Supplementary Table S5. Virulence factors were identified by type and the counts for each *Aeromonas* strain.

**Strain**	**Flp type IV pili**	**Lateral flagella**	**MSHA type IV pili**	**Polar flagella**	**Tap type IV pili**	**Type I fimbriae**	**T2SS**	**T3SS**	**T6SS**	**Toxin**	**Iron uptake**	**Other**
A. popoffii CIP 105493	0	1	17	60	16	5	14	0	22	10	9	3
A. rivuli DSM 22539	1	31	16	63	14	2	15	0	20	3	9	4
A. dhakensis AAK1	0	1	17	65	19	5	15	0	21	9	9	4
A. piscicola LMG 24783	13	32	17	71	21	4	14	37	21	6	9	4
A. molluscorum 848	0	1	17	54	12	2	14	0	0	3	9	4
A. jandaei CECT 4228	0	32	16	71	19	5	15	35	19	4	9	2
A. allosaccharophila CECT 4199	1	1	17	60	19	0	15	2	0	3	9	2
A. media WS	0	0	5	35	12	2	15	0	2	4	8	3
A. caviae CECT 838	0	32	16	63	21	2	15	0	19	3	9	3
A. schubertii strain WL1483	0	30	16	68	13	5	15	37	2	2	9	6
A. salmonicida ATCC 33658	10	32	10	66	20	5	15	5	15	5	9	4
A. australiensis CECT 8023	0	1	17	59	20	0	15	0	0	3	9	1
A. simiae CIP 107798	0	11	17	61	13	2	15	0	20	2	6	3
A. taiwanensis LMG 24683	0	32	16	65	13	2	15	0	0	3	9	4
A. lacus AE122	0	32	17	67	20	2	15	35	20	3	9	2
A. veronii CECT 4257	13	1	17	60	20	5	14	0	0	4	9	4
A. hydrophila ATCC 7966	13	1	14	65	19	5	15	0	22	12	9	4
A. eucrenophila CECT 4224	0	32	17	65	21	2	15	0	20	3	9	3
A. sobria CECT 4245	0	1	17	58	21	5	15	0	24	5	9	2
A. bestiarum CECT 4227	0	1	17	65	18	5	15	0	23	12	9	5
A. encheleia CECT 4342	0	32	16	65	18	2	15	38	20	3	9	3
A. tecta CECT 7082	0	32	17	65	19	2	15	34	20	3	9	3
A. finlandiensis 4287D	4	32	16	69	19	4	15	37	21	3	9	2
A. aquatica MX16A	0	1	17	60	22	5	15	0	22	12	9	5
A. bivalvium CECT 7113	0	31	16	63	21	2	14	0	22	3	9	4
A. enteropelogenes CECT 4487	0	32	17	70	19	0	15	0	19	5	8	5
A. diversa CECT 4254	0	32	17	69	15	0	15	34	0	5	9	1
A. fluvialis LMG 24681	0	1	17	63	19	0	15	0	1	3	9	2
A. sanarellii LMG 24682	0	1	15	65	14	2	15	0	0	3	9	3
